# Engineered phage with cell-penetrating peptides for intracellular bacterial infections

**DOI:** 10.1128/msystems.00646-23

**Published:** 2023-08-18

**Authors:** Min Zhao, Xin Tan, Zi-qiang Liu, Lei Dou, Dong Liu, Yong-jun Pan, Ying-fei Ma, Jia-lin Yu

**Affiliations:** 1 Department of Neonatology, Children’s Hospital of Chongqing Medical University, National Clinical Research Center for Child Health and Disorders, Ministry of Education Key Laboratory of Child Development and Disorders, Chongqing Key Laboratory of Child Infection and Immunity, Chongqing Key Laboratory of Pediatrics, Chongqing, China; 2 CAS Key Laboratory of Quantitative Engineering Biology, Shenzhen Institute of Synthetic Biology, Shenzhen Institute of Advanced Technology, Chinese Academy of Sciences, Shenzhen, China; 3 Department of Neonatology, Southern University of Science and Technology Hospital, Shenzhen, China; 4 Department of Neonatology, Shenzhen People’s Hospital, Shenzhen, China; 5 Department of Critical Care Medicine, Southern University of Science and Technology Hospital, Shenzhen, China; Institut François Jacob, Evry, France

**Keywords:** phage engineering, cell-penetrating peptide, phage therapy, intracellular infection

## Abstract

*Salmonella* infection poses a critical challenge to global public health, and the situation is exacerbated by the increasing prevalence of antibiotic resistance. Bacteriophages (phages) are increasingly being used as antimicrobial agents due to their ability to kill specific bacteria. However, the low cellular uptake of phages has limited their use in treating intracellular bacterial infections. Here, we present a study using engineered phages with cell-penetrating peptides (CPPs) for enhancing the internalization efficiency of phages to inhibit bacterial intracellular infections. Through bioinformatic analysis, we identified a phage-encoded protein harboring an immunoglobulin-like (Ig-like) domain as the potential target for phage display. Using a CRISPR-Cas9-based method, we successfully displayed short peptides on GP94, an Ig-like domain-containing protein, of *Salmonella* phage selz. We improved phage intracellular uptake in multiple cell types by fusion of various CPPs to GP94. Notably, the phage selz_HA-TAT_ showed promising results in enhancing the intracellular inhibition of *Salmonella* in different cells. Our research provides a straightforward strategy for displaying CPPs on non-model phages, offering a promising novel and effective therapeutic approach for treating intracellular and drug-resistant bacteria.

**IMPORTANCE:**

*Salmonella* infection is a significant threat to global public health, and the increasing prevalence of antibiotic resistance exacerbates the situation. Therefore, finding new and effective ways to combat this pathogen is essential. Phages are natural predators of bacteria and can be used as an alternative to antibiotics to kill specific bacteria, including drug-resistant strains. One significant limitation of using phages as antimicrobial agents is their low cellular uptake, which limits their effectiveness against intracellular bacterial infections. Therefore, finding ways to enhance phage uptake is crucial. Our study provides a straightforward strategy for displaying cell-penetrating peptides on non-model phages, offering a promising novel and effective therapeutic approach for treating intracellular and drug-resistant bacteria. This approach has the potential to address the global challenge of antibiotic resistance and improve public health outcomes.

## INTRODUCTION

*Salmonella* infection is a major public health concern worldwide, in both developing and developed countries, and has contributed to an increased economic burden on the health systems (1, 2). *Salmonella* is a common facultative intracellular pathogen, which can invade and proliferate in a variety of phagocytic and non-phagocytic cells including epithelial cells of the gut and gallbladder, dendritic cells, and macrophages, causing food-borne gastroenteritis or the more severe enteric or typhoid fever (3). After *Salmonella* infects the host cell, it survives and replicates within the *Salmonella*-containing vacuole (SCV). It can lyse the nascent SCV and escape into the cytosol of epithelial cells to achieve hyper-replication (4), likely leading to recurrent infections. The intracellular localization protects the pathogens from antibiotics and hosts immune responses (5), greatly increasing the difficulty of clinical treatment. Meanwhile, in recent years, the effectiveness of antimicrobial therapy for *Salmonella* infection has been threatened by the emergence and dissemination of drug-resistant strains (6). Thus, there is an urgent need for a new approach to control *Salmonella* infection.

Bacteriophages (phages) have been used as antimicrobial agents for nearly 100 years and are greatly encouraged in pre-clinical and clinical studies due to the rapid rise of antibiotic resistance (7 – 9). The advantages of phage therapy over conventional antibiotic therapy include the following: (i) minimal disruption of normal microbial flora, (ii) efficacy treatment of antibiotic-resistant infections, (iii) replication *in situ* at the site of infection, and (iv) low toxicity (10). However, the application of phages to inhibit intracellular pathogens has been greatly limited.

Previous studies have demonstrated that phages can be internalized via receptor-mediated endocytosis, and non-specific receptor-mediated mechanisms, including pinocytosis and micropinocytosis (11 – 14). However, the low efficacy of cellular entry exhibited by most phages hinders their ability to effectively kill intracellular bacteria. To enhance the efficacy of intracellular bacterial killing, phages must be efficiently transduced into eukaryotic cells. Recent studies have shown that phage M13, T4, and T7 capsid fused with cell-penetrating peptides (CPPs) through phage display can significantly increase their internalization efficiency into eukaryotic cells (15 – 18). Thus, engineering phages through this approach holds promise for treating intracellular bacterial infections. However, displaying peptides on non-model phage surfaces is challenging due to the limited knowledge of phage structure.

In this study, we engineered a non-model *Salmonella* phage that displays CPPs to inhibit intracellular *Salmonella* infections. Initially, we conducted a bioinformatic analysis to screen the phage protein candidates for peptide display from a phage genome database of the cultured phage isolates. We then used a CRISPR-Cas9-based method to display peptides on a *Salmonella* phage from our laboratory phage collection. Subsequently, we constructed phages modified with selected CPPs, evaluated the internalization efficiency of engineered phages under multiple mammalian cells, and tested their inhibitory activity against intracellular *Salmonella* with the most promising candidate in multiple mammalian cell infection models.

## RESULTS

### Screening and validation of protein candidates for peptides display

**Fig 1:**
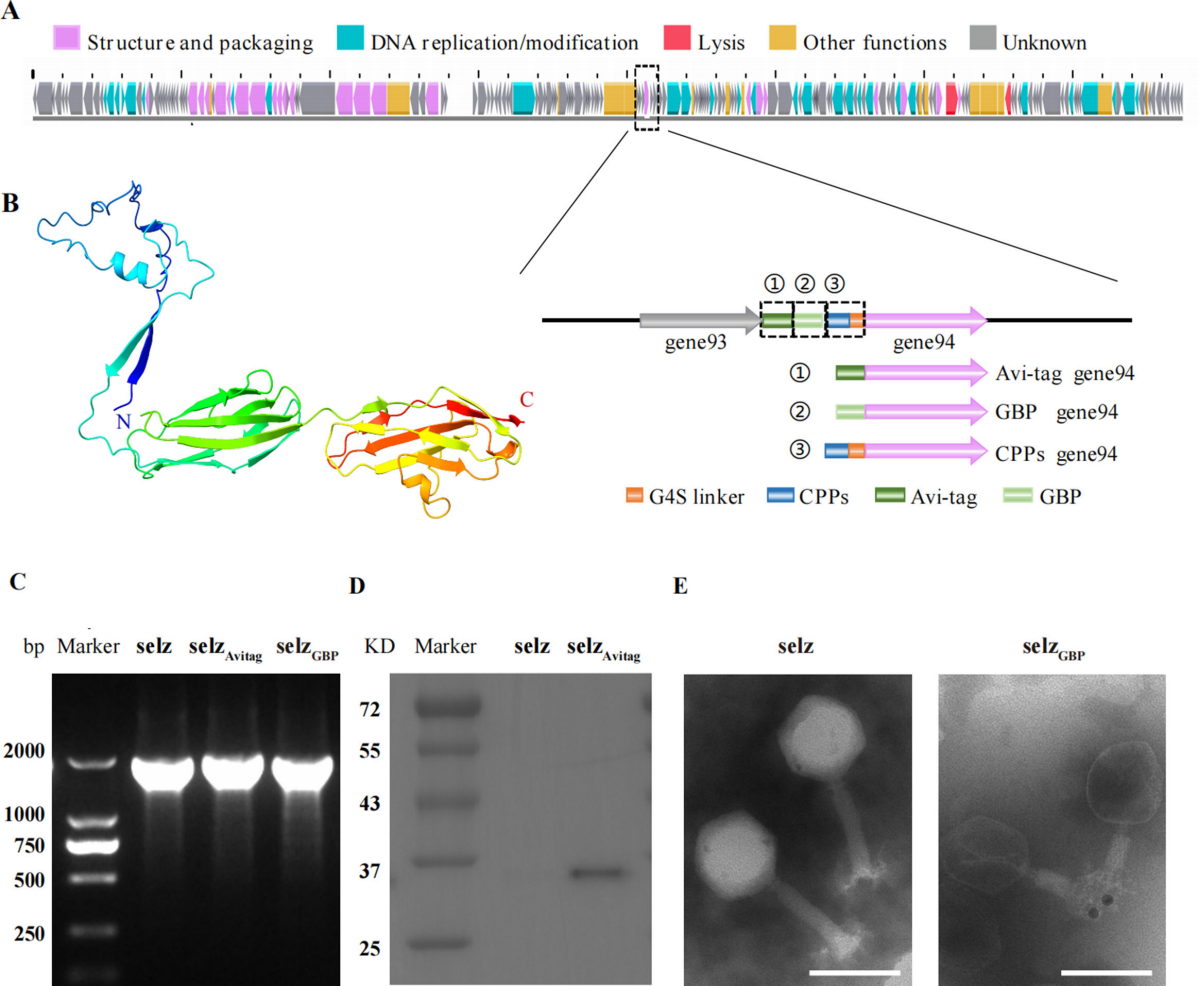
Screening and validation of phage protein candidates for peptides display. (A) Schematic of gene editing strategy in this study. Genome annotation of phage selz, the color of the open reading frames (ORFs) refers to five modules: structure and packaging, purple; host lysis, red; DNA replication and modification, light blue; other functions, yellow; and unknown, gray. GP94 is outlined by a dotted black line. Avi-tag (green) or gold-binding peptide (GBP, light green) is directly fused at the N-terminus of GP94, and CPPs (blue) are linked with a 2xG4S linker (orange) at the N-terminus of GP94 (pink). (B) Ribbon representations of the predicted structure (rainbow) of protein GP94 with AlphaFold2. (C) Identification of recombinant phage selz_avitag_ and selz_GBP_ using primers Fw94 and Rev94. (D) Western blotting on polyvinylidene difluoride(PVDF) membrane using horseradish peroxidase(HRP)-labeled streptavidin. (E) TEM images of wild-type selz phage (left) and selz_GBP_ (right) with 10 nm gold nanoparticles. Scale bar, 100 nm. TEM, transmission electron microscopy.

Recent work has shown that immunoglobulin-like (Ig-like) domains are frequently displayed on the surface of tailed dsDNA phages (19, 20). This suggests that the phage structure protein containing Ig-like domains could serve as a promising site for phage display. In this study, we analyzed our laboratory phage collection using the Pfam searching protocol (19) (see details in Materials and Methods), and we found that the phage protein GP94 of a *Salmonella* phage selz (Genbank: MH709121) contains the Ig-like domain. The structure of GP94 was further predicted with AlphaFold2 (21, 22), which contains three domains, including two Ig-like domains (Fig. 1B; Fig. S1). Comparison of this structure (with high accuracy) with the protein data bank (PDB) using the Dali server (23) shows the high similarity to three phage structure proteins: hoc protein of phage RB49, tail tube protein (pb6) of phage T5, and tail tube protein (gpV) of phage lambda (Table S1; Fig. S2). Indeed, the hoc protein of phage RB49 and gpV protein of phage lambda have been used for phage display (24, 25), suggesting that GP94 could be a promising protein for phage display.

We further fused the Avi-Tag, which can be efficiently biotinylated *in vitro*, at the N-terminus of GP94 in phage selz via a CRISPR-Cas9-based method (26). The gel electrophoresis and Sanger sequencing result confirmed that the Avi-Tag sequence was fused to the gene of GP94 (Fig. 1C). Western-blot analysis detected the signal of the Avi-Tag-labeled biotinylated GP94 protein (Fig. 1D). Additionally, we fused a GBP at the N-terminus of GP94 (27) and successfully detected the gold nanoparticles (GNPs) using TEM. GP94 protein was found on the tail of the phage with 10 nm GNPs, while no GNPs were detected in the WT phage (Fig. 1E). These findings suggest that GP94 can be applied for peptides display in further studies.

### Engineered phages maintain biological properties after fusion with CPPs

Nowadays, hundreds of CPPs are known and can be divided into three classes: protein-derived peptides, designed peptides, and model peptides (28). Integrin, as a protein-derived peptide, has revealed good internalization in Hela cells (29). The human protein-derived arginine-rich P8, as a novel CPP, has confirmed that it is capable of delivering cargoes (fluorescein isothiocyanate [FITC], peptide, and protein) into a variety of human cells and was found to be approximately 10-fold more efficient than transactivator of transcription (TAT) (30, 31). Transportan, a fusion peptide of galanin and mastoparan that has distinct hydrophobic and hydrophilic parts, was found to be the most efficient CPP among 22 sequences for delivering an organic fluorophore into mammalian cells (29). R7 and R8 are predominantly cationic, as model peptides are widely used in the literature (29, 32). Furthermore, the cyclization of CPPs has recently been shown to increase cellular uptake and promote the direct translocation of CPP-conjugated proteins into the cytosol (33). Most importantly, cyclic TAT has demonstrated to enable efficient cytosolic delivery of protein cargos (34). HA-TAT peptide has previously been used to deliver peptides and proteins into the cytosol and then trafficked to the nucleus (32, 35). To treat intracellular *Salmonella* infections, these seven different CPPs with high efficacy to transport macromolecular substances into mammalian cells were fused with GP94 in phage selz with the same strategy mentioned above (29, 31, 32) (Table 1; Fig. S3). WT selz and engineered phages produced similar translucent phage plaque with halo following 24-h incubation with *S*. Typhimurium SL1344 expressing mCherry (mCherry-SL1344) (Fig. S4A). The one-step growth curve indicated both WT and engineered phages had a 30-min latency, reached a plateau level at 50 min, and had a burst size of 57–104 phage particles per infected bacteria cell (*P* > 0.05) (Fig. S4B). Overall, the fusion of CPPs with phages did not affect their biological properties.

**TABLE 1:** CPPs amino acid sequence information^*a*^

Name	CPP sequence	Linker	References
HA-TAT	GDIMGEWGNEIFGAIAGFLGYGRKKRRQRR	GGGGSGGGGS	(32, 35)
cTAT	CYGRKKRRQRRRC	GGGGSGGGGS	(34)
Integrin	VTVLALGALAGVGVG	GGGGSGGGGS	(29)
P8	RRWRRWNRFNRRRCR	GGGGSGGGGS	(30, 31)
R8	RRRRRRRR	GGGGSGGGGS	(32)
R7	RRRRRRR	GGGGSGGGGS	(29)
Transportan	GWTLNSAGYLLGKINLKALAALAKKL	GGGGSGGGGS	(29)

^*a*^ The amino acid sequence of each CPP and the peptide linker.

### CPPs enhance the uptake of phages into mammalian cells

To investigate whether CPPs increase the uptake of phage selz into mammalian cells, multiple cells were incubated with WT selz and engineered phages at the same concentration (1.5 × 10^9^ plaque forming units, PFU) for 4 h. The intracellular active phage titer was quantified using plaque assay. We tested five cell lines, including Hela, A549, Caco-2, THP-1, and Raw264.7. Hela, Caco-2, differentiated THP-1, and Raw264.7 cells are commonly used in *Salmonella* infection model (4, 36 – 38). Furthermore, Hela cells are frequently used in CPP studies (29, 32, 39), while A549 cells are often used for phage cellular uptake studies (17, 40).

**Fig 2:**
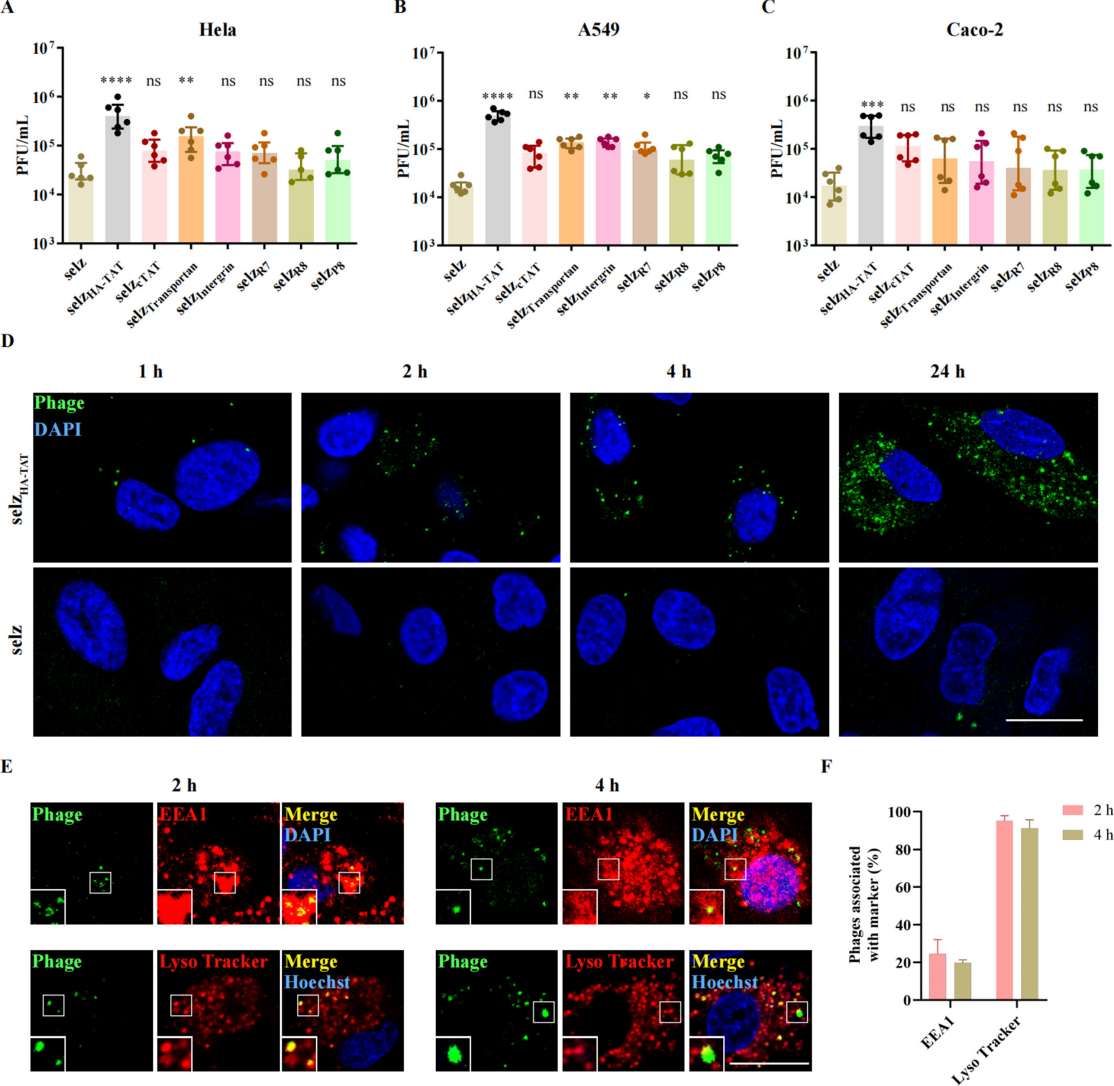
Cellular uptake of WT and engineered selz phage in different cell lines. Phages were incubated with Hela (A), A549 (B), and Caco-2 (C) cells (80%–90% confluence) at 1.5 × 10^9^ PFU for 4 h. Cells were washed with phosphate-buffered saline (PBS) buffer four times, lysed by ddH_2_O, and then functional phages were quantified using plaque assay. Data are presented as median with interquartile range (IQR) of the results from two independent experiments (*n* = 6–8, ****P < 0.0001, ***P < 0.001, *P < 0.05, the comparison was exclusively performed between individual engineered phage and WT selz phage). (D) Time course of WT selz and selz_HA-TAT_ phages internalization into A549 cells. Following incubation with AF488 NHS-labeled phages (green) for the time indicated at 37°C, the non-internalized phages were stripped off with PBS containing 0.5 mg/mL heparin sulfate. (E) Phage clusters in early-endosomal/lysosomal compartments. A549 cells were incubated with AF488 NHS-labeled selz_HA-TAT_ phages (green) for 2 h and 4 h at 37°C, extracellular phages were removed by heparin sulfate, and the fixed cells were immunostained for the early-endosomal marker [early endosome antigen 1 (EEA1)] (red), and nuclei were stained with 4',6-diamidino-2-phenylindole (DAPI) (blue). The lysosomal staining dye LysoTracker Red and cell nuclei stain Hoechst (blue) were added to the medium 20 min before the end of incubation with phage. Representative images from two to four biological replicates are shown. Scale bar, 20 μm. (F) Quantification of phage clusters positive for EEA1 or LysoTracker. Following incubation with AF488 NHS-labeled phages for 2 h or 4 h at 37°C. The data represent the mean ± standard deviation of two independent experiments depicting the results from at least 200 cells for each condition.

Engineered phages selz_HA-TAT_, selz_Transportan_, selz_Intergrin_, and selz_R7_ showed a significantly higher (*P* < 0.05) uptake number of phages than WT selz in A549 cells (Fig. 2B). Meanwhile, an increased uptake number of selz_HA-TAT_ (*P* < 0.0001) and selz_Transportan_ (*P* < 0.01) was observed in Hela cells (Fig. 2A), and an enhanced uptake number of selz_HA-TAT_ (*P* < 0.001) was also observed in Caco-2 cells (Fig. 2C). Notably, selz_HA-TAT_ showed the highest uptake number of phages (10–30 times higher than WT selz) in all tested epithelial cells. No significant difference (*P* > 0.05) in phage uptake between WT and engineered phages was observed in phagocytes (Fig. S5A and B). However, WT selz displayed a higher internalization efficacy in phagocytes than in epithelial cells.

To further confirm the phages within the intracellular space, confocal microscopy was employed using A549 cells for the most promising engineered phage, selz_HA-TAT_. The phage labeled with Alexa Fluor 488 NHS ester was incubated with cells for serial time points. The cells were treated with PBS containing 0.5 mg/mL heparin sulfate and then were observed using microscopy. According to the literature, heparin sulfate, which carries a high negative charge, can strip off the positively charged CPP-modified phages that were bound to the cell membrane (32, 41, 42). Compared with WT selz, a visibly increased green fluorescence signal was detected with selz_HA-TAT_ (Fig. 2D). For selz_HA-TAT_, after 1 h of incubation, clusters of fluorescent phages started to appear in the cell and their amount increased up to 24 h (Fig. 2D). To determine whether internalized phage particles were shuttled through the endolysosomal pathway, phage-incubated cells were stained for early endosomes (EEA1) and late endosomes/lysosomes (LysoTracker, acidotropic dye). After 2 h, a minor portion of phages was associated with early endosomes, whereas most phages were found in the late endosomal/lysosomal compartments (Fig. 2E and F). In addition, after 4 h, we observed phage clusters in lysosomal compartments in A549, Hela, and Caco-2 cells through three-dimensional (3D) reconstruction (Fig. S7). These observations confirmed that the engineered phages were taken up within intracellular space rather than attached to the cell membrane. Interestingly, after 4 h, most of the intracellular phages were still co-localized with LysoTracker (Fig. 2E and F). This observation suggested that engineered phage selz_HA-TAT_ was entrapped in endosomes. Although HA-TAT peptide has previously been reported to deliver peptides and proteins into the cytosol (35), only a small portion of phages was escaped into the cytosol environment.

These results validated previous findings, suggesting that WT phage may translocate into mammalian cells, albeit at a relatively low rate (13). Furthermore, our findings suggested that CPPs can increase intracellular phage uptake. Notably, selz_HA-TAT_ displays the highest intracellular phage uptake efficiency.

### Characterization of engineered phage selz_HA-TAT_

**Fig 3:**
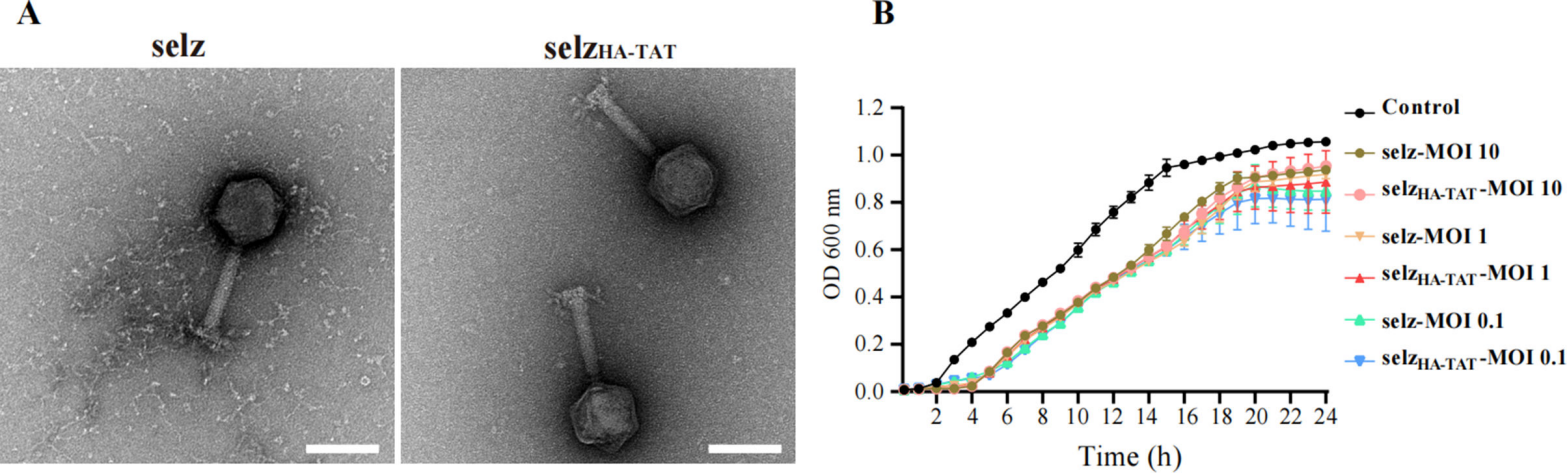
Characteristics of WT selz and selz_HA-TAT_. (A) Typical TEM images of WT selz and selz_HA-TAT_. Scale bar, 100 nm. (B) Growth curve of SL1344 with WT selz and selz_HA-TAT_ at MOI 0.1, 1, and 10. Values represent the mean with standard deviation.

Our results suggested that selz_HA-TAT_ was highly internalized in multiple epithelial cells (Fig. 2A through C), making it a promising candidate for inhibiting intracellular bacterial infections. Subsequently, we characterized this engineered phage. TEM images showed that both WT selz and selz_HA-TAT_ were approximately 86 nm wide and 95 nm long with a contractile tail measuring approximately 113 nm (Fig. 3A). No discernible differences were observed between these two phages. Moreover, both WT selz and selz_HA-TAT_ were similarly effective in inhibiting the growth of mCherry-SL1344 at multiplicity of infection (MOI) 0.1, 1, and 10. Both phages strongly suppressed the growth of mCherry-SL1344 up to 4 h, resulting in a lower plateau level compared to the no-phage control group (Fig. 3B). However, bacterial growth was observed after 4 h, which may be attributed to the emergence of phage-resistant bacteria (43).

### Engineered phage selz_HA-TAT_ inhibits intracellular bacteria

To test whether phage selz_HA-TAT_ with the highest cellular uptake efficiency can enhance the killing of intracellular *Salmonella* SL1344, we determined the intracellular killing efficacies of WT selz and selz_HA-TAT_ in three epithelial cell lines (A549, Hela, and Caco-2) with mCherry-SL1344.

**Fig 4:**
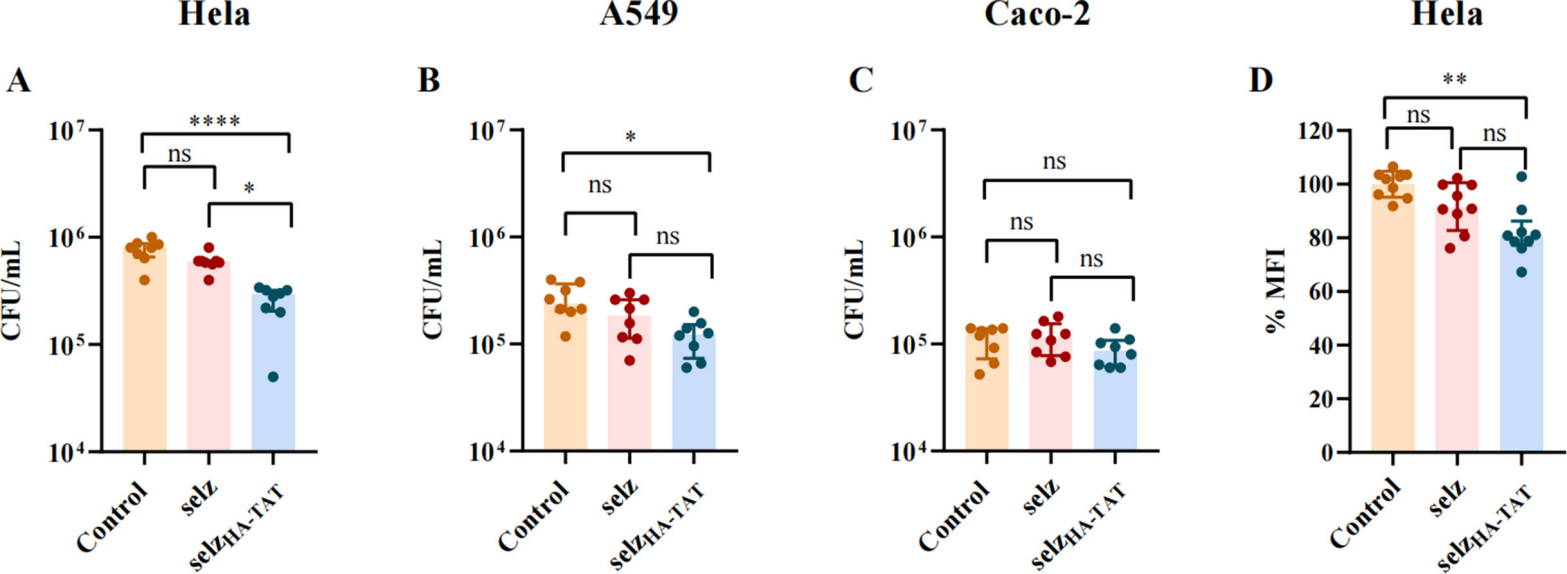
Killing efficacy of intracellular *Salmonella* by selz_HA-TAT_ on three different epithelial cell lines. Hela (A), A549 (B), and Caco-2 (C) cells were infected with mCherry-SL1344 (MOI,10) for 12 h and treated with 1.5 × 10^9^ PFU phages for 4 h. The control group received no phage treatment. *Y*-axis represents the counts of intracellular mCherry-SL1344. Values represent the median with interquartile range (IQR) of the results from two independent experiments (****P < 0.0001, *P < 0.05, and ns: no significance). (D) Mean fluorescence intensity (MFI) of mCherry-SL1344-infected Hela cells. *Y*-axis represents the ratio of MFI from the phage group to the control group. Values represent the median with IQR of the results from two independent experiments (**P < 0.01 and ns: no significance).

Cells were infected with mCherry-SL1344 at an MOI of 10 for 12 h. Residual bacteria in the medium were inactivated by the addition of gentamicin before treatment with phage. Following 4-h incubation at 37°C, the number of mCherry-SL1344 that remained in epithelial cells was quantified by bacterial counting. Compared to WT selz that showed no significant inhibition against intracellular mCherry-SL1344, phage selz_HA-TAT_ exhibited a significant decrease in the number of bacteria inside Hela (*P* < 0.0001) and A549 (*P* < 0.05) cells, with a killing efficacy of 64% and 48%, respectively (Fig. 4A and B). The inhibition of intracellular mCherry-SL1344 by phage selz_HA-TAT_ was further supported by the results of phage quantification, which showed a fivefold increase in titer compared to that of WT selz inside Hela (*P* < 0.0001) and A549 (*P* < 0.0001) cells (Fig. S8A and B). However, phage selz_HA-TAT_ showed negligible inhibition against intracellular mCherry-SL1344 in Caco-2 cells (Fig. 4C), despite an increased phage titer (*P* < 0.0001) (Fig. S7C).

To further determine the inhibition efficacy of selz_HA-TAT_, the fluorescence intensity of mCherry, which represents the survival of SL1344 inside Hela cells, was measured by flow cytometry. The MFI of Hela cells did not decrease after treatment with WT selz, while the MFI of Hela cells decreased by 18% (*P* < 0.01) after treatment with selz_HA-TAT_ (Fig. 4D). These results confirmed that phage selz_HA-TAT_ can efficiently target and kill intracellular mCherry-SL1344 bacteria.

### Engineered phage selz_HA-TAT_ is non-cytotoxic

**Fig 5:**
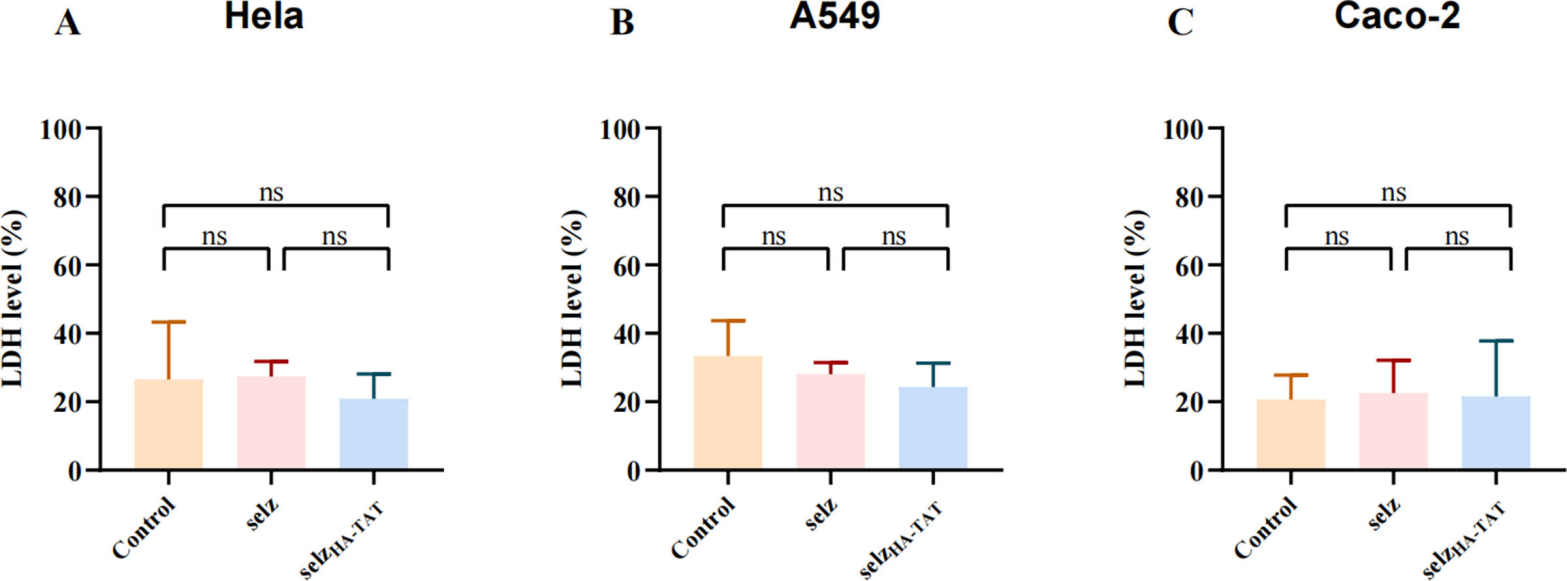
Cellular cytotoxicity of WT selz and selz_HA-TAT_. Phages (1.5 × 10^9^ PFU) were incubated with Hela (A), A549 (B), and Caco-2 (C) cells for 4 h, and lactate dehydrogenase (LDH) levels were measured. Analysis was performed with the Pierce LDH cytotoxicity assay. Data represent the results of the median with interquartile range from two independent experiments (ns, no significance).

Agents that are capable of translocating into mammalian cells must be evaluated for their potential cytotoxic effects (44). To assess the safety of selz_HA-TAT_ on mammalian cells, we conducted cytotoxicity assays using multiple cell types. Following 4-h exposure, no evidence of cytotoxicity was observed (Fig. 5). Therefore, we concluded that selz_HA-TAT_ is a safe and viable option for treating intracellular infections *in vitro.*

## DISCUSSION

Currently, the work of expression exogenous peptide has mainly occurred to the model phages of *Escherichia coli* phages T7, T4, lambda, and M13 (15, 16, 25, 45). It is difficult to directly express foreign peptides on non-model phage surfaces due to the lack of phage structure information. Although several studies reported the expression of exogenous proteins on non-model phages, these works were performed on phages with a high sequence similarity to the model phage (46, 47).

The Ig-like fold, which is one of the most common and widely dispersed folds in nature, comprises at least seven β-strands arranged into two distinct sheets packed in a parallel manner (19). Proteins possessing the Ig-like fold are commonly found in bacteria and are most often involved in cell-cell adhesion or extracellular glycohydrolysis (19). Several works indicated that phages can also interact with mucus in mammalian organisms via these displayed Ig-like domains (20, 48), which suggested that these Ig-like domains were exposed on the surface of the phage. In this study, we demonstrated that a *Salmonella* phage protein with Ig-like domains can display short peptides (Fig. 1B, D, and E; Table S1).

Transcytosis of phages has been considered a general mechanism for phage traversing across the epithelial lumens, which may account for this huge phage population in the human body (13). Researchers have attempted to exploit this phage feature to target intracellular bacterial infections and got some encouraging results (46, 49). For example, lytic phage vB_SauM_JS25 was able to penetrate bovine mammary epithelial cells and effectively kill intracellular *Staphylococcus aureus* (49). In another study, an engineered T7-like phage K1F-GFP efficiently inhibited intracellular *E. coli* after entering human urinary bladder epithelial cells (46). Moreover, chemical modification of phage surface or encapsulation phage in liposomes can further increase phage internalization and enhance the inhibition of intracellular bacterial infection (50, 51). Notably, phages modified to display CPPs on their surface were also found to promote their internalization into mammalian cells (15 – 17). CPPs-modified phages have been used as nanovectors for gene delivery and vaccination, with T4 phage heads decorated with TAT (HIV-1-trans-activating peptide) showing increased gene delivery into various mammalian cells and inducing a robust immune response in mice (15). In this study, we utilized CPPs-displayed phages to target intracellular bacterial infections. Our results were consistent with previous studies, indicating that CPPs-displayed phage significantly enhanced phage uptake (Fig. 2A through C) and increased inhibition of intracellular infection (Fig. 4A, B, and D). Although the increased killing of intracellular bacteria was significant, it was mild (Fig. 4D), possibly due to the nature of phage selz (Fig. 3B). Interestingly, no obvious inhibition of intracellular bacteria was observed in Caco-2 cells (Fig. 4C). Previous studies indicated that the majority of *Salmonella* reside in the cytosol of epithelial cells 8 h post-infection; however, S*almonella* exhibited different infectivity and cytosolic replication characteristics across various epithelial cell lines (52). More intracellular and cytosolic bacteria were found in Hela cells compared with Caco-2 cells under the same condition (52). Similarly, fewer intracellular bacteria were observed in Caco-2 cells compared with Hela and A549 cells in this study (Fig. 4). In addition, a similar titer of intracellular engineered phages was observed in these cells (Fig. S8). According to our analysis, the inhibition of intracellular bacteria by the engineered phage can be attributed to the eradication of cytosolic bacteria. This is supported by the fact that the majority of *Salmonella* bacteria reside in the cytosol of epithelial cells at 8 h post-infection (52). The observed absence of apparent inhibition of intracellular bacteria in Caco-2 cells, upon exposure to the engineered phages, is likely due to the relatively low abundance of cytosolic bacteria being targeted. Consequently, fewer bacteria are affected, leading to a limited inhibitory effect.

Since CPP was discovered in 1988, it has been commonly used as a molecular transporter. CPP has shown great potential in medical applications due to its high internalization capacity and low toxicity (53, 54). In general, a drug tagged with a CPP resulted in higher amounts at the target site when compared with the non-tagged drug (51). Toward this aspect, a direct fusion of a CPP to vancomycin has been shown to lead to increased intracellular killing *in vitro* and improved pharmacokinetics *in vivo* (55). Furthermore, a cleavable conjugate of a CPP with kanamycin also significantly reduced intracellular *Salmonella* number *in vitro* and in a *Caenorhabditis elegans* model (56). Although the results of these studies are highly encouraging, they still rely on classical antibiotics for bacterial killing, which contributes little to the fight against the growing crisis of antibiotic resistance. In addition, phages offer the unique advantage of high efficacy against antibiotic-resistant bacteria. For these reasons, CPP-fused phages hold promise as novel and effective future therapeutics.

Indeed, phage particles and CPPs induce different types of endocytosis by the recognition of distinct cell surface receptors; there might exist complex interactions among these different endocytic pathways (14, 16, 40). There are studies suggesting that CPPs-induced endocytosis played a major role during the phage uptake process (16, 57), which is the case in these epithelial cell lines as well (Fig. 2E and F).

Given the limited knowledge of the potential of CPPs-modified phages for intracellular delivery, our work could provide useful suggestions for the choice of CPPs. Although the CPPs we chose could significantly increase intracellular small molecules delivery in the previous study (29, 31, 32), only HA-TAT-fused phages showed significantly enhanced intracellular uptake of active phages in three epithelial cells including Hela, A549, and Caco-2 (Fig. 2A through C). No significant increase of phage uptake with CPPs was observed in these phagocytes (Fig. S5). The cellular uptake of CPPs depends on various factors including label or cargo, concentration of CPP, cell type, stage of cell cycle, incubation time and temperature, etc. (58). Here, the engineered phages with CPPs showed altered penetration was probably due to the influence of the large size of phage particles and different experimental conditions. Phagocytes are the main components of innate immunity, and pathogens and phages can be internalized and cleared from the host by phagocytic cells via phagocytosis (59, 60). Therefore, CPPs likely have a limited effect on phage internalization within phagocytes, leading to an insufficient efficacy of phage internalization with phagocytes that already exhibit a robust capacity for phage internalization. Although the high internalization and efficient endosomal escape capacity of HA-TAT (35) were reported, it fails to deliver phage particles efficiently into the cytosol environment (Fig. 2E and F). The HA-TAT sequence may have a small positive effect on endosomal escape. However, its efficacy may be insufficient to facilitate escape when dealing with large-sized phage particles. It is noteworthy that selz_HA-TAT_ shows no noticeable cytotoxicity in mammalian cells (Fig. 5).

It is important to note that this study was limited to *in vitro* experiments, and further research is needed to determine the efficacy and safety of this approach *in vivo*. Additionally, although we observed enhanced phage internalization and inhibition of intracellular bacteria, this strategy can be further optimized. No significant increase in penetration into phagocytic cells was observed for these engineered phages compared to WT phages (Fig. S5). Phagocytic cells are vital reservoirs for intracellular pathogens (61, 62). It is necessary to conduct further research into additional CPPs to identify the specific ones that have the potential to enhance phage penetration into phagocytes, promising effective elimination of intracellular infections. Furthermore, the majority of internalized phages were entrapped in the late endosomal/lysosome compartment (Fig. 2E and F). To enhance phage endosomal escape and consequently increase intracellular bacteria killing, it is worth exploring additional variations of CPPs, including multivalent CPPs and fusion CPPs with pH-dependent membrane-active peptides (63). Moreover, the occurrence of phage resistance was observed at 4 h *in vitro* study (Fig. 3B). To address this challenge, an evolutionary study can be conducted in future investigations to identify phage mutants with enhanced lytic activity to counteract the development of resistance and improve the effectiveness of bacteria killing (64).

In conclusion, this study provides proof-of-concept evidence that no-model phages can be engineered to display CPPs with enhanced cellular uptake, potentially leading to improved bacterial killing. This approach has the potential to address the growing issue of antibiotic resistance and warrants further investigation.

## MATERIALS AND METHODS

### Phage Ig-like domains analysis in phage genomes

To seed our search for phage Ig-like domains, we searched the laboratory *Salmonella* phage collection using the hmmscan command in HMMER3 (*e*-value cutoff set to 1e−5) (65) against a wide variety of Pfam sequence alignment database entries corresponding to domains classified as Ig-like beta-sandwich folds (Table S3) in the Pfam.v.35 database (66).

### Protein structure prediction and alignment

The structure prediction software AlphaFold2 (21) was applied for the structure prediction of protein GP94. We used the ColabFold notebook, whose structure prediction is powered by AlphaFold2 combined with a fast, multiple sequence alignment generation stage using MMseqs2 (22). The final predicted domain structures were submitted to the Dali server to identify the closest structural homologs in the PDB (23). Views of the domain 3D structures were prepared with ChimeraX. The protein structure alignment was performed by the RSCB.org web portal (67, 68).

### Bacteria, phage, plasmids, and media

*S.* Typhimurium strain SL1344 containing plasmid pBBR1MCS-Tac-mCherry (mCherry-SL1344) was kindly provided by Shanghai Jiao Tong University. The *Salmonella* phage selz (Genbank: MH709121) and *S.* Typhimurium model strain SL7207 are obtained from our laboratory. Phage selz is a member of the myovirus (Ackermannviridae family), and it can efficiently infect *S.* Typhimurium SL1344 and SL7207. All phages for confocal image and cell treatment were purified by CsCl density gradient ultracentrifugation (with three solutions in densities of 1.7, 1.5, and 1.3 g/mL) and then dialyzed with molecular weight cutoff of 50 kDa dialysis tube. And the titer was tested through double agar overlay plaque assays.

We adapted the type II CRISPR/Cas9 system from *Streptococcus pyogenes* for phage genome editing (26). The plasmids pDonor and spCas9 were obtained from the laboratory of Chenli Liu, Shenzhen Institute of Synthetic Biology. The backbone of the spCas9 plasmid was amplified from the pCas plasmid (Addgene plasmid id: 62225). The plasmid of spCas9 consists of Cas9, lambda-red, an antibiotic-resistant gene, and the SacB gene. The temperature-sensitive replicon of spCas9 has two-point mutations (9,697 and 10,395, A–G) compared with that of pCas. The backbone of the pDonor plasmid was amplified from the plasmid (Addgene plasmid id: 62226) with the modifications of a chloramphenicol-resistant gene (Cm*^R^*) and spacer. The spCas9 plasmid was transformed into SL7207 grown in Luria-Bertani (LB) broth supplemented with 50 µg/mL kanamycin to generate the SL7207 (spCas9) strains. LB containing 1.5% agar was used as a solid medium.

The phage genome was examined using sgRNAcas9 V3.0 (69) to identify the possible protospacer sequences, and the most suitable protospacers were selected. The DNA fragments of the plasmid pDonor, two 500 bp homologous arms, AviTag, GBP, CPPs, G4S linker, and sgRNA were amplified using polymerase chain reaction (PCR) (Table S2; 2× Phanta Max Master Mix, Vazyme, P525). The recombination module contained homologous arms, functional peptides, and sgRNA sequences obtained by overlapping PCR. Then recombinant plasmids were obtained by using the ClonExpress II One Step Cloning Kit (Vazyme, C112-01) to connect the pDonor backbone and the recombination module.

### Generation of engineered phage

Both plasmids of recombinant pDonor and spCas9 were transformed into SL7207. Then WT phages were propagated on SL7207, harboring the appropriate pDonor and spCas9 for three rounds. The last round of phage lysate was appropriately diluted and mixed with 200 µL of mid-log-phase SL7207 without plasmid, then mixed with 5 mL of molten 0.5% soft agarose, and immediately poured onto LB plates for single-phage plaque formation. Randomly selected single plaques dissolved in 50 µL saline magnesium (SM) buffer, then 0.5 µL of this buffer was used as a template in PCR reactions using primers seq94fw and seq94rev. The final recombinant phages were obtained by three purifications and confirmed via colony PCR and Sanger sequencing. All the primer sequences used in this study were ordered from BGI (Beijing Genomics Institute) and are shown in Table S2.

### Phage characterization assay

To detect whether there is a difference between the general characteristics of engineered phages and WT phages, we test the plaque morphology, one-step growth assay, growth curves, and size measurement. The plaque morphology of selz and engineered phages was tested on SL1344 through double agar overlay plaque assay. Briefly, 10 µL dilution of phage suspension and 100 µL overnight host bacteria liquid cultures were mixed with 5 mL 0.5% soft LB agar, then poured into the lower layer of 1.5% solid LB agar plate for incubation at 37°C overnight.

The one-step growth assay was tested as described previously (26). Briefly, 2 mL of log-phase (optical density [OD_600_] = 0.5–0.6) host bacteria was mixed with phage suspension at an MOI of 0.01. After co-incubation for 5 min at 37°C, 220 rpm, the mixture was centrifuged at 13,000 rpm for 1 min to remove non-adsorbed phages and then 2 mL preheated fresh LB liquid was added and incubated at 37°C with shaking. PFU of the supernatant was determined by spot testing from 0 to 1 h with 10-min intervals. Each experiment was performed three times.

Growth curves of selz_HA-TAT_ and WT phage were performed as previously reported (26). The log-phase host strain was diluted to 10^5^ CFU/mL, and then the phage suspension was added at an MOI of 0.1, 1, and 10. Then, 200 µL mixture was added to a 96-well tissue culture plate. Each group performed in three replicates. LB liquid group and host bacteria without phage served as a negative and positive group, respectively. The plate was incubated at 37°C continuous oscillation culture, and the OD_600_ value was measured every 10 min for 24 h in a microplate reader.

### Western-blot analysis

Phage selz_avitag_ biotinylation was carried out by Biotin Labeling Kit for Avi-tag Protein with BirA (Beyotime, P0630M). Phages were propagated with SL1344 at an MOI of 0.1 overnight, and then 10% PEG8000 and 1 mol/L NaCl were added to the phage supernatant overnight at 4°C. Subsequently, the phage pellet was recovered and dissolved in PBS buffer after centrifugation at 8,300 rpm at 4°C for 10 min, and then 10% chloroform was added to extract bacterial fragments and PEG8000. Further purification was achieved by CsCl density gradient ultracentrifugation to obtain high purity and high concentration phages. Then 300-kDa dialysis tube was used to remove excess extract debris. Biotinylation of phages was performed according to the instructions, with biotin ligase buffer A (10×) 5 µL, biotin ligase buffer B (10×) 5 µL, BirA (100×) 1 µL, and 10^10^–10^12^ phage particles to 50 µL, reacting at 30°C for 2 h. Finally, dialysis was performed in a 50-kDa dialysis membrane for 16–20 h. After boiling 10 min with 5× SDS buffer, selz and selz_avitag_ were analyzed on 12.5% SDS-polyacrylamide gel electrophoresis, then electrotransferred to a PVDF membrane and using 5% bovine serum albumin blocking 2 h at room temperature. Subsequent incubation with HRP-labeled streptavidin (Sangon Biotech, B110053) (1:2,000) for 1 h at 37°C was performed to detect biotinylation of GP94 protein using Avi-Tag. After repeated washing with TBST three times, mix enhanced chemiluminescence (ECL) developer in a dark place in a 1:1 ratio, completely cover the PVDF membrane, and perform protein development at the UVP ChemStudio 515 (Analytik Jena) 3 min later.

### Transmission electron microscopy observation

To observe phage morphology, 20 µL of 10^12^ PFU/mL selz and selz_HA-TAT_ stock solution was added to the copper mesh and precipitated for 20 min. Then, 10 µL of 2% phosphotungstic acid (PTA) was dropped to stain the samples. FEI tecnai G2 spirit twin transmission electron microscope was used to observe the morphology of phages.

The selz_GBP_ and selz phage purified by CsCl density gradient ultracentrifugation were mixed with 10 nm GNP in a 1:10 ratio for a few hours at room temperature. Nickel electron microscope grids with thin film carbon supports were glow discharged and placed carbon side down in 20 µL of the phage mixture solution for 20 min. Subsequently, they were washed three times in PBS and then negatively stained with 10 µL of 2% PTA for 1 min. Grids were then visualized using a Tungsten filament HITACH HT7700 transmission electron microscope at 80 kV.

### Tissue cell culture

In this study, three different cell lines were adopted, including epithelial cell lines, Hela (cervical adenocarcinoma cells), A549 (human tumorigenic lung epithelial cell line), Caco-2 (colorectal adenocarcinoma cells), phagocyte cell lines, Raw264.7 (mouse monocyte leukemia cells), and THP-1 (human monocyte leukemia-induced macrophages), which were kindly supplied by Group Xian-en Zhang, Shenzhen Institute of Advanced Technology, Chinese Academy of Sciences. THP-1 was cultured in 1640 media containing 0.05 mM 2-mercaptoethanol, supplemented with 10% fetal bovine serum (FBS) (Gibco), and 100 µg/mL penicillin-streptomycin (PS) and then 200 ng/mL phorbol myristate acetate was added for induced THP-1 to macrophage. The other cells were cultured with Dulbecco's Modified Eagle Medium (DMEM) supplemented with 10% FBS and 100 µg/mL PS.

### Phage internalization assay

We compared the intracellular uptake efficiency of phage selz and seven different CPPs-modified phages in five mammalian cells (Hela, A549, Caco-2, Raw264.7, and THP-1). Cells were seeded on a 12-well plate at a density of 1 × 10^5^ cells grown 2–3 days to confluence. Phages at a titer of 1.5 × 10^9^ PFU/mL were co-incubated with these cells for 4 h at 37°C in a humidified 5% CO_2_ incubator. Then the cell layers were rinsed three to four times with PBS to completely remove the extracellular phage; subsequently, the intracellular phages were released by ddH_2_O lysing the cells. Phage enumeration of lysate suspension was carried out using plaque assays.

### Immunofluorescence and colocalization analysis

Phage particles were labeled with Alexa Fluor 488 NHS Ester (Yeasen, 40779ES03) (50 µg for ~10^11^ PFU) for 2 h at room temperature in dark and shaken once every 15 min. Then unbound dye was removed by PBS, and then NHS-AF488-labeled phages were mixed with mCherry-SL1344 and observed by confocal microscope to confirm phage successful labeling (Fig. S6). Two milliliters containing 1.5 × 10^9^ PFU/mL selz and selz_HA-TAT_ phage labeled NHS-AF488 was added in 35 × 35 mm glass bottom microscope dishes with cell concentration of approximately 80% confluence and cultivated for 1, 2, 4, and 24 h, respectively. To stain lysosomes, live cells were incubated with the nucleic acid dye Hoechst and LysoTracker Red (Invitrogen, L7528) for 20 min before observation. Cells were washed three times with 0.5 mg/mL heparin sulfate solution (Sigma, H3149) and twice with PBS to remove the extracellular phages. Meanwhile, followed by washing three times with heparin sulfate, cells were fixed by 4% paraformaldehyde, immunofluorescence staining was performed with the early-endosomal marker anti-EEA1 (rabbit IgG, abcam, ab109110) and subsequently reacted with secondary antibody anti-rabbit IgG (H + L), F(ab')2 Fragment Alexa Fluor 647 Conjugate (CST, 4414) and finally, nucleic acid staining was performed with DAPI. Samples are stored at 4°C protected from light.

The 3D reconstruction using a *z* stack to visualize the intracellular localization of phages was performed on three epithelial cells at 4 h. For the quantification of phage clusters positive for EEA1 or LysoTracker, at least 200 cells were identified and scored phage-positive vesicles for each experiment. Confocal images were acquired with a 60× oil-immersion objective (NIS-Elements, Nikon).

### Cytotoxicity assay

To test the toxicity of cells following phage internalization, an LDH assay was adopted. Cells (2 × 10^4^) were seeded in 96-well microtiter plates and cultivated to 80% confluence. After rinsing with PBS twice, 200 µL containing 1.5 × 10^9^ PFU/mL phage was added. Subsequently, cells were incubated in 5% CO_2_ at 37℃ for 4 h. Two percent Triton X-100 was applied as the positive control, PBS treatment as the negative control, and cell culture medium without cell as background. Fifty microliters of 10-fold diluted supernatant and an equal volume of LDH reaction mixture were mixed in a black 96-well plate and incubated at room temperature for 1 h. Then the luminescent signal was detected by a fluorescence microplate reader (BioTek, Synergy H1). Cytotoxicity was calculated using the formula: (experimental group value-background value)/(positive group-background value) × 100.

### Intracellular bacterial survival assay

The *Salmonella* intracellular infection model was performed as described previously (50). Cells (5 × 10^4^) were seeded in 12-well microtiter plates. These cells were used for infection within 24 h. Cells were infected with mCherry-SL1344 at a multiplicity of infection of 10 for 12 h and washed three times with PBS containing 100 µg/mL gentamicin to remove extracellular bacteria. Cells were then incubated in a medium with gentamicin (10 µg/mL). Subsequently, cells were treated with WT phage and selz_HA-TAT_ (1.5 × 10^9^ PFU/mL) for 4 h. The cells were washed three to four times with PBS and then ddH_2_O water was added for lysing the cell. Then the suspension was serially diluted in SM buffer. The internalized phages and bacteria were counted by colony-forming and plaque assays.

### Flow cytometric analysis

After phage treatment for 4 h, the cell layers were rinsed once with PBS and then 0.25% trypsin was added to digest cells for 5 min. The cell culture medium is then added to resuspend the cells. They are centrifuged at 800 rpm for 3 min to obtain the cell pellet. After repeated washing three times with 4°C pre-chilled PBS, add PBS resuspended on ice, and then analyzed the fluorescence values with a flow cytometer (Beckman Coulter, CytoFLEX S).

### Statistical analysis

Kruskal-Wallis with Dunn’s test was used to evaluate the significant difference among multiple groups, and Mann-Whitney test was used for two-group comparisons. All statistical analyses were performed using Prism 7.04 (GraphPad, San Diego, CA, USA), and differences with *P* < 0.05 were considered statistically significant.

## SUPPLEMENTAL MATERIAL

Supplemental material msystems.00646-23-s0001.pdf Figures S1 to S8 and Tables S1 to S3.

ASM does not own the copyrights to Supplemental Material that may be linked to, or accessed through, an article. The authors have granted ASM a non-exclusive, world-wide license to publish the Supplemental Material files. Please contact the corresponding author directly for reuse.

## References

[1] Ehuwa O , Jaiswal AK , Jaiswal SS . 2021. Food safety and food handling practices. Foods 10:907. doi:10.3390/foods10050907 33919142 PMC8143179

[2] Galán JE . 2021. Salmonella Typhimurium and inflammation: a pathogen-centric affair. Nat Rev Microbiol 19:716–725. doi:10.1038/s41579-021-00561-4 34012042 PMC9350856

[3] Ibarra JA , Steele-Mortimer O . 2009. Salmonella – the ultimate insider. Salmonella virulence factors that modulate intracellular survival. Cell Microbiol 11:1579–1586. doi:10.1111/j.1462-5822.2009.01368.x 19775254 PMC2774479

[4] Malik-Kale P , Winfree S , Steele-Mortimer O . 2012. The bimodal lifestyle of intracellular Salmonella in epithelial cells: replication in the cytosol obscures defects in vacuolar replication. PLoS One 7:e38732. doi:10.1371/journal.pone.0038732 22719929 PMC3374820

[5] Petit TJP , Lebreton A . 2022. Adaptations of intracellular bacteria to vacuolar or cytosolic niches. Trends in Microbiology 30:736–748. doi:10.1016/j.tim.2022.01.015 35168833

[6] da Silva KE , Tanmoy AM , Pragasam AK , Iqbal J , Sajib MSI , Mutreja A , Veeraraghavan B , Tamrakar D , Qamar FN , Dougan G , Bogoch I , Seidman JC , Shakya J , Vaidya K , Carey ME , Shrestha R , Irfan S , Baker S , Luby SP , Cao Y , Dyson ZA , Garrett DO , John J , Kang G , Hooda Y , Saha SK , Saha S , Andrews JR . 2022. The international and intercontinental spread and expansion of antimicrobial-resistant Salmonella Typhi: a genomic epidemiology study. Lancet Microbe 3:e567–e577. doi:10.1016/S2666-5247(22)00093-3 35750070 PMC9329132

[7] Tan X , Chen H , Zhang M , Zhao Y , Jiang Y , Liu X , Huang W , Ma Y . 2021. Clinical experience of personalized phage therapy against carbapenem-resistant Acinetobacter baumannii lung infection in a patient with chronic obstructive pulmonary disease. Front Cell Infect Microbiol 11:631585. doi:10.3389/fcimb.2021.631585 33718279 PMC7952606

[8] Marongiu L , Burkard M , Lauer UM , Hoelzle LE , Venturelli S . 2022. Reassessment of historical clinical trials supports the effectiveness of phage therapy. Clin Microbiol Rev 35:e0006222. doi:10.1128/cmr.00062-22 36069758 PMC9769689

[9] Boucher D , Barnich N . 2022. Phage therapy against adherent-invasive E. coli: towards a promising treatment of Crohn’s disease patients J Crohns Colitis 16:1509–1510. doi:10.1093/ecco-jcc/jjac070 35796668

[10] Venturini C , Petrovic Fabijan A , Fajardo Lubian A , Barbirz S , Iredell J . 2022. Biological foundations of successful bacteriophage therapy. EMBO Mol Med 14:e12435. doi:10.15252/emmm.202012435 35620963 PMC9260219

[11] Bodner K , Melkonian AL , Covert MW . 2021. The enemy of my enemy: new insights regarding bacteriophage-mammalian cell interactions. Trends Microbiol 29:528–541. doi:10.1016/j.tim.2020.10.014 33243546

[12] Lehti TA , Pajunen MI , Skog MS , Finne J . 2017. Internalization of a polysialic acid-binding Escherichia coli bacteriophage into eukaryotic neuroblastoma cells. Nat Commun 8:1915. doi:10.1038/s41467-017-02057-3 29203765 PMC5715158

[13] Kormelink TG , Meijer IC . 2018. What clicks actually mean: exploring digital news user practices. Journalism 19:668–683. doi:10.1177/1464884916688290 29782573 PMC5944087

[14] Miernikiewicz P , Dąbrowska K . 2022. Endocytosis of bacteriophages. Curr Opin Virol 52:229–235. doi:10.1016/j.coviro.2021.12.009 34968792

[15] Tao P , Mahalingam M , Marasa BS , Zhang Z , Chopra AK , Rao VB . 2013. In vitro and in vivo delivery of genes and proteins using the bacteriophage T4 DNA packaging machine. Proc Natl Acad Sci U S A 110:5846–5851. doi:10.1073/pnas.1300867110 23530211 PMC3625312

[16] Kim A , Shin T-H , Shin S-M , Pham CD , Choi D-K , Kwon M-H , Kim Y-S . 2012. Cellular Internalization mechanism and intracellular trafficking of filamentous M13 phages displaying a cell-penetrating transbody and TAT peptide. PLoS One 7:e51813. doi:10.1371/journal.pone.0051813 23251631 PMC3522607

[17] Grigonyte AM , Hapeshi A , Constantinidou C , Millard A . 2021. Modification of bacteriophages to increase their association with lung epithelium cells in vitro. Pharmaceuticals 14:308. doi:10.3390/ph14040308 33915737 PMC8067280

[18] Xu H , Bao X , Wang Y , Xu Y , Deng B , Lu Y , Hou J . 2018. Engineering T7 bacteriophage as a potential DNA vaccine targeting delivery vector. Virol J 15:49. doi:10.1186/s12985-018-0955-1 29558962 PMC5859711

[19] Fraser JS , Yu Z , Maxwell KL , Davidson AR . 2006. Ig-like domains on bacteriophages: a tale of promiscuity and deceit. J Mol Biol 359:496–507. doi:10.1016/j.jmb.2006.03.043 16631788

[20] Barr JJ , Auro R , Furlan M , Whiteson KL , Erb ML , Pogliano J , Stotland A , Wolkowicz R , Cutting AS , Doran KS , Salamon P , Youle M , Rohwer F . 2013. Bacteriophage adhering to mucus provide a non-host-derived immunity. Proc Natl Acad Sci U S A 110:10771–10776. doi:10.1073/pnas.1305923110 23690590 PMC3696810

[21] Jumper J , Evans R , Pritzel A , Green T , Figurnov M , Ronneberger O , Tunyasuvunakool K , Bates R , Žídek A , Potapenko A , Bridgland A , Meyer C , Kohl SAA , Ballard AJ , Cowie A , Romera-Paredes B , Nikolov S , Jain R , Adler J , Back T , Petersen S , Reiman D , Clancy E , Zielinski M , Steinegger M , Pacholska M , Berghammer T , Bodenstein S , Silver D , Vinyals O , Senior AW , Kavukcuoglu K , Kohli P , Hassabis D . 2021. Highly accurate protein structure prediction with Alphafold. Nature 596:583–589. doi:10.1038/s41586-021-03819-2 34265844 PMC8371605

[22] Mirdita M , Schütze K , Moriwaki Y , Heo L , Ovchinnikov S , Steinegger M . 2022. Colabfold: making protein folding accessible to all. Nat Methods 19:679–682. doi:10.1038/s41592-022-01488-1 35637307 PMC9184281

[23] Holm L . 2020. Using dali for protein structure comparison. Methods Mol Biol 2112:29–42. doi:10.1007/978-1-0716-0270-6_3 32006276

[24] Fokine A , Islam MZ , Zhang Z , Bowman VD , Rao VB , Rossmann MG . 2011. Structure of the three N-terminal immunoglobulin domains of the highly Immunogenic outer capsid protein from a T4-like bacteriophage▿. J Virol 85:8141–8148. doi:10.1128/JVI.00847-11 21632759 PMC3147960

[25] Pavoni E , Vaccaro P , D’Alessio V , De Santis R , Minenkova O . 2013. Simultaneous display of two large proteins on the head and tail of bacteriophage Lambda. BMC Biotechnol 13:79. doi:10.1186/1472-6750-13-79 24073829 PMC3850075

[26] Yuan S , Shi J , Jiang J , Ma Y . 2022. Genome-scale top-down strategy to generate viable genome-reduced phages. Nucleic Acids Res 50:13183–13197. doi:10.1093/nar/gkac1168 36511873 PMC9825161

[27] Hou J , Xu Y , Sun S , Zhong X , Yang C-T , Zhou X . 2023. Gold nanoparticles-decorated M13 phage SPR probe for dual detection of antigen biomarkers in serum. Sens Actuators B Chem 374:132811. doi:10.1016/j.snb.2022.132811

[28] Zorko M , Langel U . 2005. Cell-penetrating peptides: mechanism and kinetics of cargo delivery. Adv Drug Deliv Rev 57:529–545. doi:10.1016/j.addr.2004.10.010 15722162

[29] Mueller J , Kretzschmar I , Volkmer R , Boisguerin P . 2008. Comparison of cellular uptake using 22 CPPs in 4 different cell lines. Bioconjug Chem 19:2363–2374. doi:10.1021/bc800194e 19053306

[30] Gautam A , Sharma M , Vir P , Chaudhary K , Kapoor P , Kumar R , Nath SK , Raghava GPS . 2015. Identification and characterization of novel protein-derived arginine-rich cell-penetrating peptides. Eur J Pharm Biopharm 89:93–106. doi:10.1016/j.ejpb.2014.11.020 25459448

[31] Gautam A , Nanda JS , Samuel JS , Kumari M , Priyanka P , Bedi G , Nath SK , Mittal G , Khatri N , Raghava GPS . 2016. Topical delivery of protein and peptide using novel cell penetrating peptide IMT-P8. Sci Rep 6:26278. doi:10.1038/srep26278 27189051 PMC4870705

[32] Patel SG , Sayers EJ , He L , Narayan R , Williams TL , Mills EM , Allemann RK , Luk LYP , Jones AT , Tsai Y-H . 2019. Cell-penetrating peptide sequence and modification dependent uptake and subcellular distribution of green florescent protein in different cell lines. Sci Rep 9:6298. doi:10.1038/s41598-019-42456-8 31000738 PMC6472342

[33] Lättig-Tünnemann G , Prinz M , Hoffmann D , Behlke J , Palm-Apergi C , Morano I , Herce HD , Cardoso MC . 2011. Backbone rigidity and static presentation of guanidinium groups increases cellular uptake of arginine-rich cell-penetrating peptides. Nat Commun 2:453. doi:10.1038/ncomms1459 21878907 PMC3265364

[34] Nischan N , Herce HD , Natale F , Bohlke N , Budisa N , Cardoso MC , Hackenberger CPR . 2015. Covalent attachment of cyclic TAT peptides to GFP results in protein delivery into live cells with immediate bioavailability. Angew Chem Int Ed Engl 54:1950–1953. doi:10.1002/anie.201410006 25521313

[35] Wadia JS , Stan RV , Dowdy SF . 2004. Transducible TAT-HA fusogenic peptide enhances escape of TAT-fusion proteins after lipid raft macropinocytosis. Nat Med 10:310–315. doi:10.1038/nm996 14770178

[36] Wrande M , Vestö K , Puiac Banesaru S , Anwar N , Nordfjell J , Liu L , McInerney GM , Rhen M . 2020. Replication of Salmonella enterica serovar Typhimurium in RAW264.7 phagocytes correlates with hypoxia and lack of iNOS expression. Front Cell Infect Microbiol 10:537782. doi:10.3389/fcimb.2020.537782 33330118 PMC7734562

[37] Luk CH , Valenzuela C , Gil M , Swistak L , Bomme P , Chang Y-Y , Mallet A , Enninga J . 2021. Salmonella enters a dormant state within human epithelial cells for persistent infection. PLoS Pathog 17:e1009550. doi:10.1371/journal.ppat.1009550 33930101 PMC8115778

[38] Knodler LA , Vallance BA , Celli J , Winfree S , Hansen B , Montero M , Steele-Mortimer O . 2010. Dissemination of invasive Salmonella via bacterial-induced extrusion of mucosal epithelia. Proc Natl Acad Sci U S A 107:17733–17738. doi:10.1073/pnas.1006098107 20876119 PMC2955089

[39] Richard JP , Melikov K , Brooks H , Prevot P , Lebleu B , Chernomordik LV . 2005. Cellular uptake of unconjugated TAT peptide involves clathrin-dependent endocytosis and heparan sulfate receptors. J Biol Chem 280:15300–15306. doi:10.1074/jbc.M401604200 15687490

[40] Bichet MC , Chin WH , Richards W , Lin Y-W , Avellaneda-Franco L , Hernandez CA , Oddo A , Chernyavskiy O , Hilsenstein V , Neild A , Li J , Voelcker NH , Patwa R , Barr JJ . 2021. Bacteriophage uptake by mammalian cell layers represents a potential sink that may impact phage therapy. iScience 24:102287. doi:10.1016/j.isci.2021.102287 33855278 PMC8024918

[41] Jones AT , Sayers EJ . 2012. Cell entry of cell penetrating peptides: tales of tails wagging dogs. J Control Release 161:582–591. doi:10.1016/j.jconrel.2012.04.003 22516088

[42] Mandal S , Mann G , Satish G , Brik A . 2021. Enhanced live-cell delivery of synthetic proteins assisted by cell-penetrating peptides fused to DABCYL. Angew Chem Int Ed Engl 60:7333–7343. doi:10.1002/anie.202016208 33615660 PMC8048964

[43] Fang Q , Feng Y , McNally A , Zong Z . 2022. Characterization of phage resistance and phages capable of intestinal decolonization of carbapenem-resistant klebsiella pneumoniae in mice. Commun Biol 5:48. doi:10.1038/s42003-022-03001-y 35027665 PMC8758719

[44] Röhrig C , Huemer M , Lorgé D , Luterbacher S , Phothaworn P , Schefer C , Sobieraj AM , Zinsli LV , Mairpady Shambat S , Leimer N , Keller AP , Eichenseher F , Shen Y , Korbsrisate S , Zinkernagel AS , Loessner MJ , Schmelcher M . 2020. Targeting hidden pathogens: cell-penetrating enzybiotics eradicate intracellular drug-resistant Staphylococcus aureus. mBio 11:e00209-20. doi:10.1128/mBio.00209-20 32291298 PMC7157818

[45] Kim G , Kim M , Lee Y , Byun JW , Hwang DW , Lee M . 2020. Systemic delivery of microRNA-21 antisense oligonucleotides to the brain using T7-peptide decorated exosomes. J Control Release 317:273–281. doi:10.1016/j.jconrel.2019.11.009 31730913

[46] Møller-Olsen C , Ho SFS , Shukla RD , Feher T , Sagona AP . 2018. Engineered K1F bacteriophages kill intracellular Escherichia coli K1 in human epithelial cells. Sci Rep 8:17559. doi:10.1038/s41598-018-35859-6 30510202 PMC6277420

[47] Oda M , Morita M , Unno H , Tanji Y . 2004. Rapid detection of Escherichia coli O157:H7 by using green fluorescent protein-labeled PP01 bacteriophage. Appl Environ Microbiol 70:527–534. doi:10.1128/AEM.70.1.527-534.2004 14711684 PMC321238

[48] Barr JJ , Auro R , Sam-Soon N , Kassegne S , Peters G , Bonilla N , Hatay M , Mourtada S , Bailey B , Youle M , Felts B , Baljon A , Nulton J , Salamon P , Rohwer F . 2015. Subdiffusive motion of bacteriophage in mucosal surfaces increases the frequency of bacterial encounters. Proc Natl Acad Sci U S A 112:13675–13680. doi:10.1073/pnas.1508355112 26483471 PMC4640763

[49] Zhang L , Sun L , Wei R , Gao Q , He T , Xu C , Liu X , Wang R . 2017. Intracellular Staphylococcus aureus control by virulent bacteriophages within MAC-T bovine mammary epithelial cells. Antimicrob Agents Chemother 61:e01990-16. doi:10.1128/AAC.01990-16 27919889 PMC5278684

[50] Meng L , Yang F , Pang Y , Cao Z , Wu F , Yan D , Liu J . 2022. Nanocapping-enabled charge reversal generates cell-enterable endosomal-escapable bacteriophages for intracellular pathogen inhibition. Sci Adv 8:eabq2005. doi:10.1126/sciadv.abq2005 35857522 PMC11581130

[51] Yan W , Banerjee P , Xu M , Mukhopadhyay S , Ip M , Carrigy NB , Lechuga-Ballesteros D , To KKW , Leung SSY . 2021. Formulation strategies for bacteriophages to target intracellular bacterial pathogens. Adv Drug Deliv Rev 176:113864. doi:10.1016/j.addr.2021.113864 34271022

[52] Knodler LA , Nair V , Steele-Mortimer O . 2014. Quantitative assessment of cytosolic Salmonella in epithelial cells. PLoS One 9:e84681. doi:10.1371/journal.pone.0084681 24400108 PMC3882239

[53] Wang F , Wang Y , Zhang X , Zhang W , Guo S , Jin F . 2014. Recent progress of cell-penetrating peptides as new carriers for intracellular cargo delivery. J Control Release 174:126–136. doi:10.1016/j.jconrel.2013.11.020 24291335

[54] Xie J , Bi Y , Zhang H , Dong S , Teng L , Lee RJ , Yang Z . 2020. Cell-penetrating peptides in diagnosis and treatment of human diseases: from preclinical research to clinical application. Front Pharmacol 11:697. doi:10.3389/fphar.2020.00697 32508641 PMC7251059

[55] Ruczyński J , Rusiecka I , Turecka K , Kozłowska A , Alenowicz M , Gągało I , Kawiak A , Rekowski P , Waleron K , Kocić I . 2019. Transportan 10 improves the pharmacokinetics and pharmacodynamics of vancomycin. Sci Rep 9:18270. doi:10.1038/s41598-019-54823-6 30824786 PMC6397271

[56] Brezden A , Mohamed MF , Nepal M , Harwood JS , Kuriakose J , Seleem MN , Chmielewski J . 2016. Dual targeting of intracellular pathogenic bacteria with a cleavable conjugate of kanamycin and an antibacterial cell-penetrating peptide. J Am Chem Soc 138:10945–10949. doi:10.1021/jacs.6b04831 27494027 PMC5482217

[57] Bhattarai SR , Yoo SY , Lee S-W , Dean D . 2012. Engineered phage-based therapeutic materials inhibit chlamydia trachomatis intracellular infection. Biomaterials 33:5166–5174. doi:10.1016/j.biomaterials.2012.03.054 22494890 PMC3341514

[58] Madani F , Lindberg S , Langel U , Futaki S , Gräslund A . 2011. Mechanisms of cellular uptake of cell-penetrating peptides. J Biophys 2011:414729. doi:10.1155/2011/414729 21687343 PMC3103903

[59] Jończyk-Matysiak E , Weber-Dąbrowska B , Owczarek B , Międzybrodzki R , Łusiak-Szelachowska M , Łodej N , Górski A . 2017. Phage-phagocyte interactions and their implications for phage application as therapeutics. Viruses 9:150. doi:10.3390/v9060150 28613272 PMC5489797

[60] Van Belleghem JD , Dąbrowska K , Vaneechoutte M , Barr JJ , Bollyky PL . 2018. Interactions between bacteriophage, bacteria, and the mammalian immune system. Viruses 11:10. doi:10.3390/v11010010 30585199 PMC6356784

[61] Kaufmann SHE , Dorhoi A . 2016. Molecular determinants in phagocyte-bacteria interactions. Immunity 44:476–491. doi:10.1016/j.immuni.2016.02.014 26982355

[62] Kellermann M , Scharte F , Hensel M . 2021. Manipulation of host cell organelles by intracellular pathogens. Int J Mol Sci 22:6484. doi:10.3390/ijms22126484 34204285 PMC8235465

[63] Erazo-Oliveras A , Muthukrishnan N , Baker R , Wang T-Y , Pellois J-P . 2012. Improving the endosomal escape of cell-penetrating peptides and their cargos: strategies and challenges. Pharmaceuticals (Basel) 5:1177–1209. doi:10.3390/ph5111177 24223492 PMC3816665

[64] Burrowes BH , Molineux IJ , Fralick JA . 2019. Directed in vitro evolution of therapeutic bacteriophages: the appelmans protocol. Viruses 11:241. doi:10.3390/v11030241 30862096 PMC6466182

[65] Eddy SR . 2011. Accelerated profile HMM searches. PLoS Comput Biol 7:e1002195. doi:10.1371/journal.pcbi.1002195 22039361 PMC3197634

[66] Mistry J , Chuguransky S , Williams L , Qureshi M , Salazar GA , Sonnhammer ELL , Tosatto SCE , Paladin L , Raj S , Richardson LJ , Finn RD , Bateman A . 2021. Pfam: the protein families database in 2021. Nucleic Acids Res 49:D412–D419. doi:10.1093/nar/gkaa913 33125078 PMC7779014

[67] Josephi B , O’Donnell P . 2023. The blurring line between freelance journalists and self-employed media workers. Journalism (Lond) 24:139–156. doi:10.1177/14648849221086806 36777088 PMC9902803

[68] Berman HM , Westbrook J , Feng Z , Gilliland G , Bhat TN , Weissig H , Shindyalov IN , Bourne PE . 2000. The protein data bank. Nucleic Acids Res. 28:235–242. doi:10.1093/nar/28.1.235 10592235 PMC102472

[69] Xie S , Shen B , Zhang C , Huang X , Zhang Y , Khodursky AB . 2014. SgRNAcas9: a software package for designing CRISPR sgRNA and evaluating potential off-target cleavage sites. PLoS ONE 9:e100448. doi:10.1371/journal.pone.0100448 24956386 PMC4067335

