# Supplementary material for: Engineered phage with cell-penetrating peptides for intracellular bacterial infections
Source: mSystems. 2023 Aug 18;8(5):e00646-23. doi: 10.1128/msystems.00646-23 (PMC10654057; doi:10.1128/msystems.00646-23)
Supplement: Supplemental material — Figures S1 to S8 and Tables S1 to S3. [file msystems.00646-23-s0001.pdf]

Supplementary Materials

Title: Engineered phage with cell-penetrating peptides for intracellular bacterial infections

Min Zhao[1], Xin Tan[2*], Zi-qiang Liu[2], Lei Dou[3], Dong Liu[4], Yong-jun Pan[5], Ying-fei Ma[2*], Jia-lin Yu[1*]

1 Department of Neonatology, Children's Hospital of Chongqing Medical University, National Clinical Research Center for Child Health and Disorders, Ministry of Education Key Laboratory of Child Development and Disorders, Chongqing Key Laboratory of Child Infection and Immunity, Chongqing Key Laboratory of Pediatrics, Chongqing, China.

2 CAS Key Laboratory of Quantitative Engineering Biology, Shenzhen Institute of Synthetic Biology, Shenzhen Institute of Advanced Technology, Chinese Academy of Sciences, Shenzhen, China

3 Department of Neonatology, Southern University of Science and Technology Hospital, Shenzhen, China

4 Department of Neonatology, Shenzhen People’s Hospital, Shenzhen, China

5 Department of Critical Care Medicine, Southern University of Science and Technology Hospital, Shenzhen, China

Correspondence:

Xin Tan, xin.tan@siat.ac.cn

Yingfei Ma, yingfei.ma@siat.ac.cn

Jia-lin Yu, yujialin486@126.com

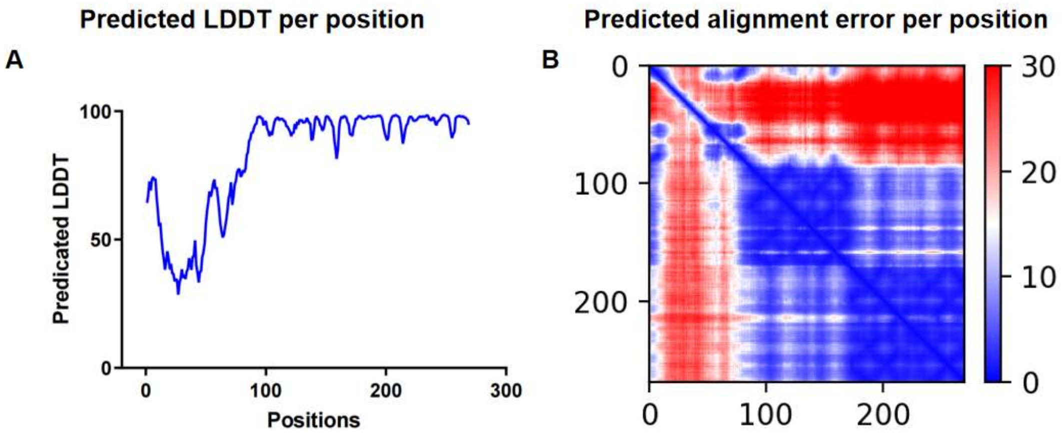

Figure S1. Predicted Local Distance Difference Test (LDDT) and alignment error per position of the 3D structure of GP94 generated by AlphaFold2. (A) The predicted LDDT scores of the predicted structure by AlphaFold2. These scores are a per-residue measure of how confident AlphaFold2 is about its prediction. (B) The predicted alignment error, which can be used to interpret the relative position of domains. A low predicted alignment error (blue) between the residues of different domains indicates that AlphaFold2 predicts the relative positions of these domains well. When the predicted alignment error between domains is high (red), the relative position of these domains is uncertain.

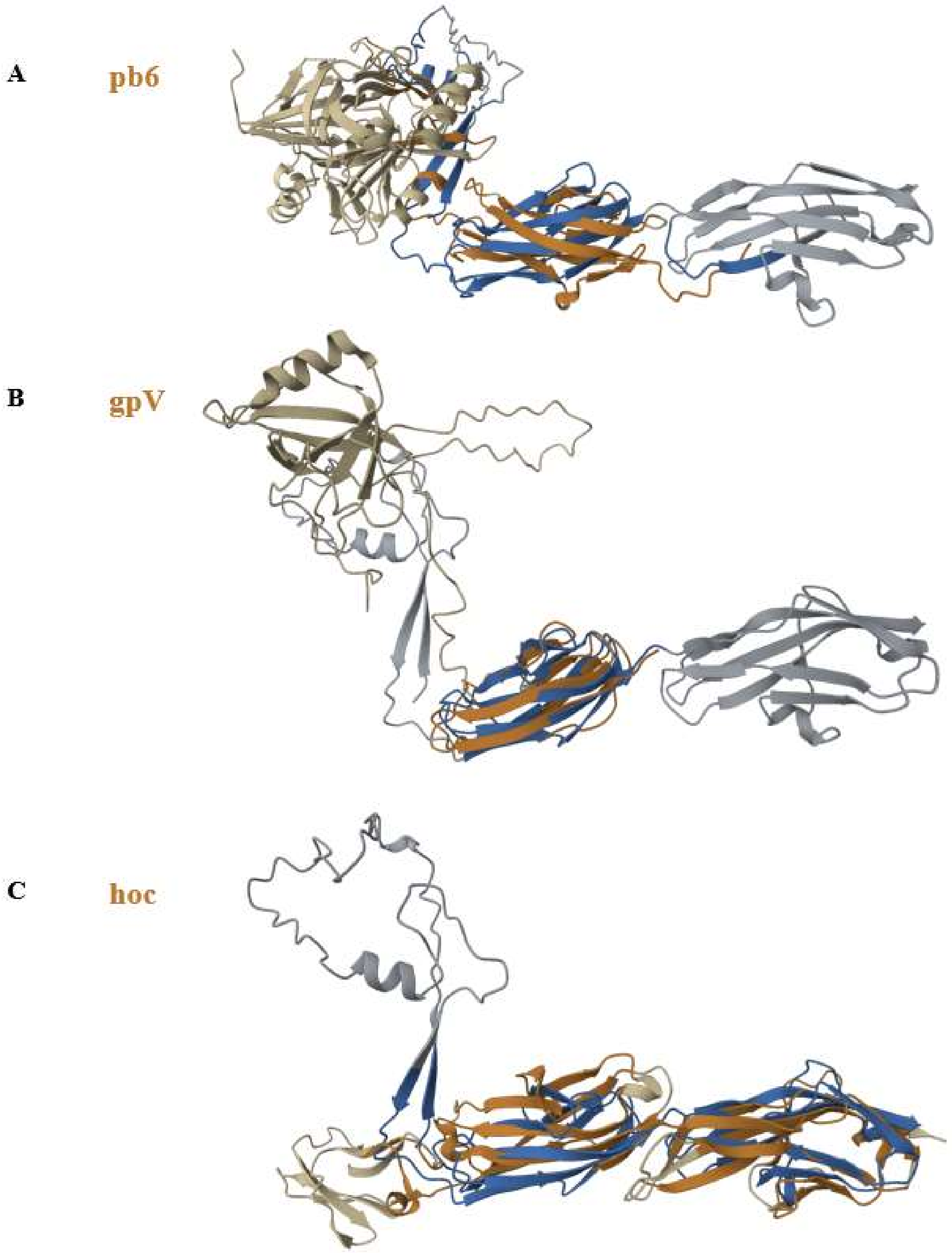

Figure S2. Structure alignment of the GP94 and its similar protein. Structure alignment of protein GP94 (blue) with tail tube protein (pb6) of phage T5 (A), tail tube protein (gpV) of phage lambda (B), and hoc protein of phage RB49 (C). GP94 structure is colored in blue and the other three protein structures are colored in orange. The no-aligned structure is colored in grey for all proteins. The figure was created via the RCSB.org web portal.

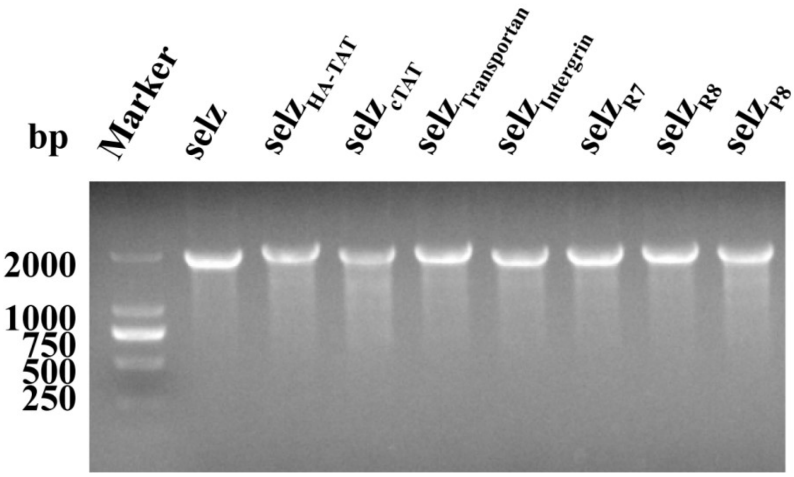

Figure S3. Identification of recombinant CPP modified phage using primers Fw94 and Rev94. M, DNA marker.

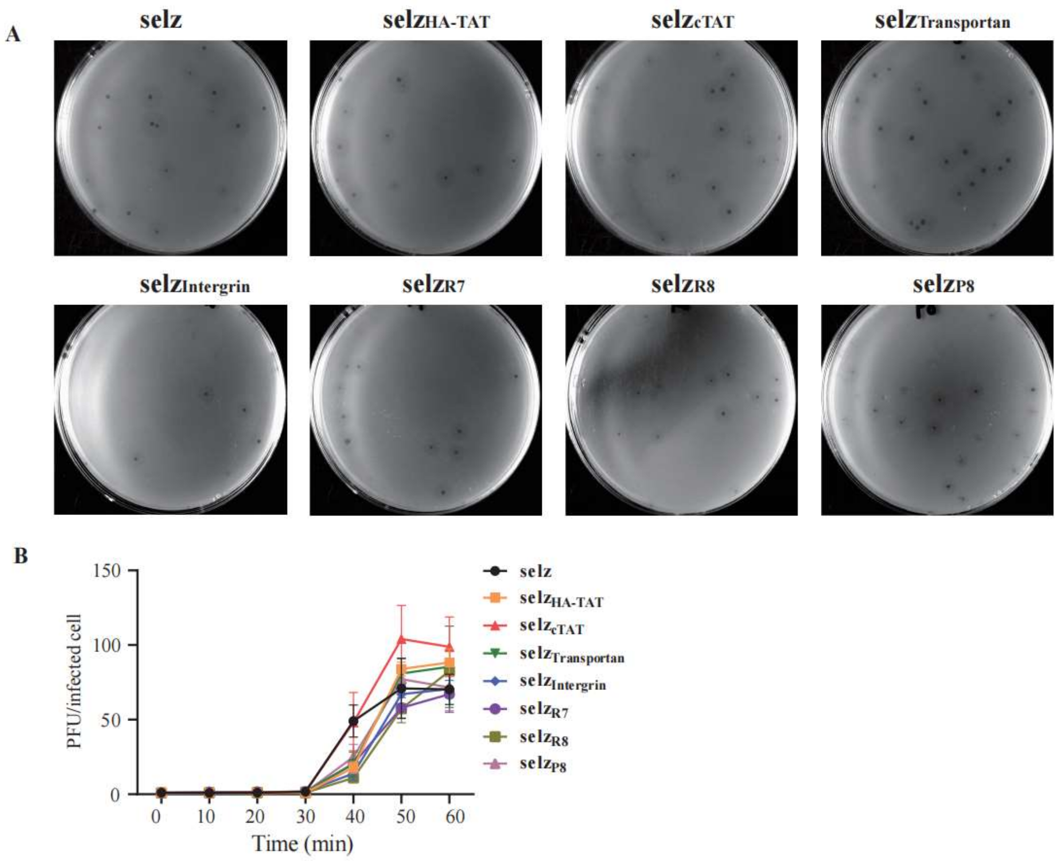

Figure S4. Characteristics of the WT selz and CPP modified phages. (A) Plaque morphology of WT selz and CPP modified phages on a bacterial lawn of SL1344. (B) The one-step growth curve of WT selz and engineered phages (n=3). Values represent the mean with standard deviation.

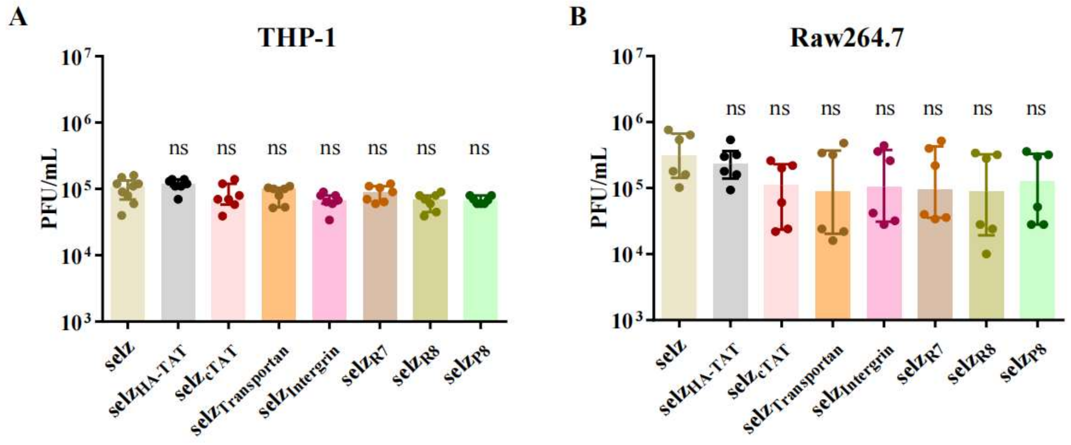

Figure S5. Cellular uptake of WT and engineered selz phage in different cell lines. Phages were incubated with THP-1 (A) and Raw264.7 cells (80–90% confluence) at 1.5*10^9 PFU for 4 h. Cells were washed with phosphate-buffered saline (PBS) buffer four times, then lysed by ddH_2O, then functional phages were quantified using plaque assay. Data are presented as median with interquartile range (IQR) of the results from two independent experiments (n = 6-8, ns, no significance, the comparison was exclusively performed between individual engineered phage and WT selz phage).

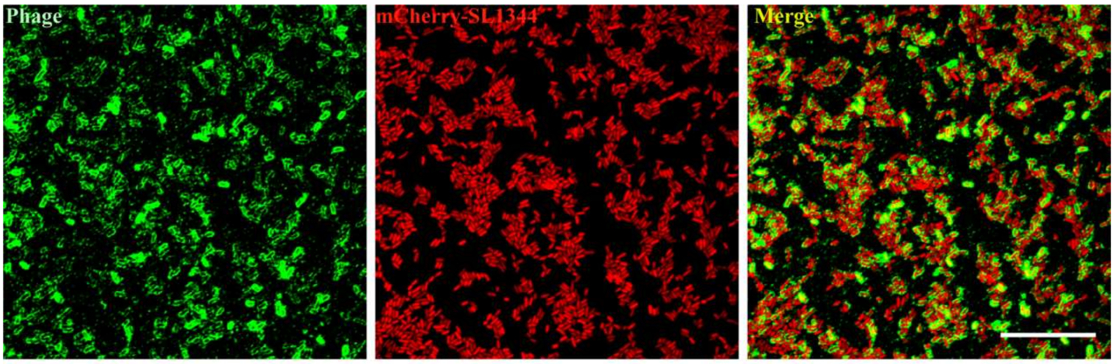

Figure S6. Confocal images of NHS-AF488 labeled phage particles (green) with mcheery-SL1344 (red). Scale bar, 20 μm.

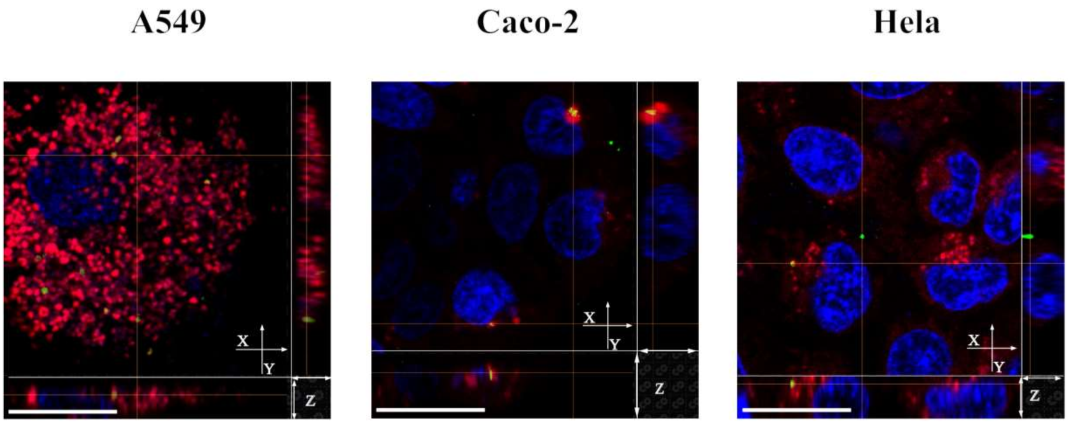

Figure S7. 3D reconstruction of confocal images. A549, Caco-2, and Hela cells were incubated for 4 h with NHS-AF488 labeled selz$_{HA\text{-}TAT}$ phages on 35*35 mm glass bottom microscope dishes before live acquisition of a high-resolution z stack to visualize phage dispersion inside of cells. The lysosomal stain LysoTracker (red) and cell nuclei stain Hoechst (blue) were added to the medium 20 min before the end of incubation with phage. The cross in the center of the image shows a cluster of internalized phages with its Z dimension (depth) represented in the side views. Scale bar, 20 μm.

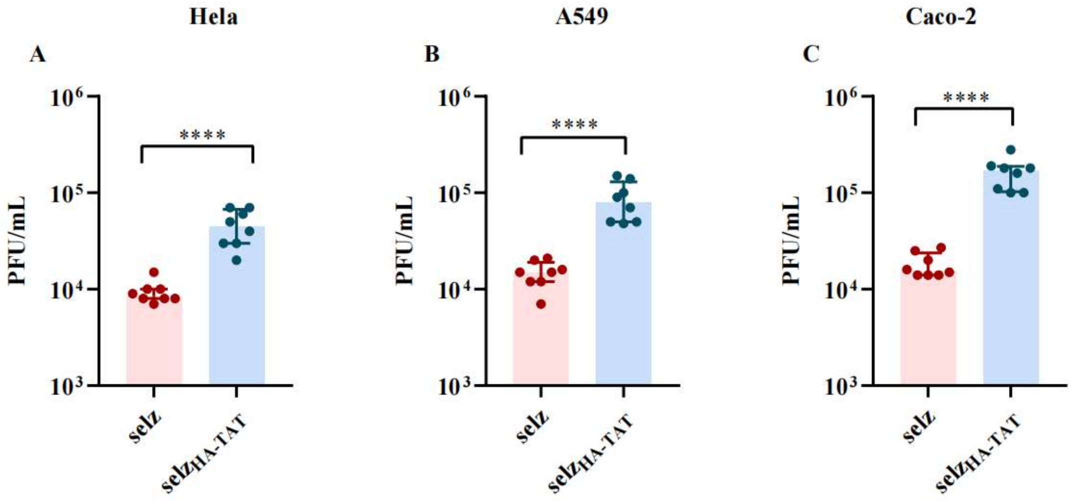

Figure S8. Intracellular phage viability of phage WT selz and selz$_{\text{HA-TAT}}$ in *Salmonella* infected cells. Hela (A), A549 (B), Caco-2 (C) cells were infected with mCherry-SL1344 (MOI,10) for 12 h and treated with $1.5*10^9$ PFU phages for 4 h. Y axis represents the number of functional phages. Values represent the median with IQR of the results from two independent experiments (*****P*<0.0001).

Domain 2					
PBD	Z-score	rmsd	lali	% id	Protein (phage)
5NGJ	11.3	1.9	80	18	Tail tube protein, pb6 (T5)
6P3E	8.3	2.7	78	24	Tail tube protein, gpV (lambda)
3SHS	4.4	3.0	75	12	Head outer capsid protein (RB49)
Domain 3					
PBD	Z-score	rmsd	lali	% id	Protein
3SHS	9.4	1.8	79	23	Head outer capsid protein (RB49)
5NGJ	5.1	2.7	73	15	Tail tube protein, pb6 (T5)

Table S1. Result of the DALI query on GP94 domain 2 (residue 92-171) and 3 (residue 172-269). Only show results related with phage proteins. rmsd: root-mean-square deviation of Cα atoms in the least-squares superimposition of the structurally equivalent Cα atoms; lali: number of structurally equivalent residues; % id: percentage of identical amino acids over all structurally equivalent residues.

Table S2. Primer used in this study

Primer names	Sequence(5‘-3’）
seq_94_FW	GCGCCGTTACGGGTTAGAT
seq_94_RV	ACGTATCACGTTCACGAGCA
seq_pXTSZT1_FW	TTCGTAAGCCATTTCCGCTCG
seq_pXTSZT1_RV	TCGGTGGTGATAAACTTATCATCCC
N20_pXTSZT1_FW	TATCCCTAGGTCTAGGGCGGC
N20_pXTSZT1_RV	ACTTGCTATTTCTAGCTCTAAAAC
pXTSZT1_FW	GTTTTAGAGCTAGAAATAGCAAGT
pXTSZT1_RV	GCCGCCCTAGACCTAGGGATA
CPP_linker_FW	GAAACCGTGTCAGCAGTCTCAATAC
HU_94_FW	TATCCCTAGGTCTAGGGCGGCGGATCCAAGAACCCCTGGGTATCGACG
HU_94_RV	CTAGTATTGAGACTGCTGACACGGTTTCCTCCACTCATCCAAATCGT
94_cpp_P2	GGGTTTGTTGCGGACAACTTCAGGGGCAACCAGAACGGTAATTGTTGGCATACTACC TCCTCCTCCACTACCTCCTCCTCCG
CPP_linker_RV	GTTTCGGGTTTGTTGCGGACAAC
HD_94_FW	GAAGTTGTCCGCAACAAACCC
HD_94_RV	CCTAGGACTGAGCTAGCTGTCAAGGTTGTCAACGACAACAGTCCTG
HD_N1_P1	ACTTGCTATTTCTAGCTCTAAAACGTGCGACTAATACTGTGATTCTTGTGATTATACC TAGGACTGAGCTAGCTGT
94_HA_T_P1	CCGTGTCAGCAGTCTCAATACTAGTCTATGGGTGATATTATGGGTGAATGGGGTAAT
94_HA_T_P2	CCGTAACCCAGAAAACCGGCAATCGCACCAAAGATTTCATTACCCCATTCACCCATAA
94_HA_T_P3	CCGGTTTTCTGGGTTACGGTCGTAAAAAACGTCGTCAGCGTCGTCGCGGAGGAGGAG GTAGTGGA
94_HA_T_P4	CCCAGAAAACCGGCAATCGCACCAAAGATTTCATTACCCCATTCACCCATAATATCAC CCATAGACTAGTATTGAGACTGCTGACACGG
94_cTAT_P1	CGTTTTTTACGACCGTAGCACATAGACTAGTATTGAGACTGCTGACACGGTTTC
94_cTAT_P2	TGTGCTACGGTCGTAAAAAACGTCGTCAGCGTCGTCGTTGCGGAGGAGGAGGTAGTG GA
94_cTAT_P3	TGTGCTACGGTCGTAAAAAACGTCGTCAGCGTCGTCGTTGCGGAGGAGGAGGTAGTA TG
94_IG_P1	AACTCCAACACCAGCCAGAGCTCCCAGCGCCAGCACGGTCACCATAGACTAGTATTG AGACTGCTGACACGGTTTC
94_IG_P2	GCTCTGGCTGGTGTTGGAGTTGGCGGAGGAGGAGGTAGTGGAGGA
94_P8_P1	GGCAACGACGACGGTTAAAACGGTTCCAACGACGCCAACGACGCATAGACTAGTATT GAGACTGCTGACACGGTTTC
94_P8_P2	CGTTTTAACCGTCGTCGTTGCCGCGGAGGAGGAGGTAGTGGAGGA
94_R7_R8_P1	CGACGACGACGACGACGACGCATAGACTAGTATTGAGACTGCTGACACGGTTTC
94_R7_P2	TCGTCGTCGTCGTCGTCGTCGCGGAGGAGGAGGTAGTGGAGGA
94_R8_P2	TCGTCGTCGTCGTCGTCGCGGAGGAGGAGGTAGTGGAGGA
94_TP_P1	GCGCTTTCAGGTTAATTTTGCCCAGCAGATAGCCCGCGCTGTTCAGGGTCCAGCCCAT AGACTAGTATTGAGACTGCTGACACGGTTTC
94_TP_P2	GGGCAAAATTAACCTGAAAGCGCTGGCGGCGCTGGCGAAAAAACTCGGAGGAGGAG GTAGTGGAGGA
94_BAP_P1	GAAACCGTGTCAGCAGTCTCAATACTAGTCTATGGGTCTTAACGACATCTTCGAGGCA CAGAAGATCGAGTGGC
94_BAP_P2	GGGTTTGTTGCGGACAACTTCAGGGGCAACCAGAACGGTAATTGTTGGCGAAAGACC CTCGTGCCACTCGATCTTCTGTGCCT
94_GBP_P1	GAAACCGTGTCAGCAGTCTCAATACTAGTCTATGGTATCAGGTTCATCACCTGACTCA ATGCCAACAATTACCGT
94_GBP_P2	GGGTTTGTTGCGGACAACTTCAGGGGCAACCAGAACGGTAATTGTTGGCATTGAGTC

Table S3. Pfam families used to search for Ig-like domains in phage genomes.

Pfam accession	Pfam full name
PF18683	Chitinase W immunoglobulin-like domain
PF05688	Bacterial Immunoglobulin-like 21
PF16706	Izumo-like Immunoglobulin domain
PF18589	Obesity receptor immunoglobulin like domain
PF18214	STATa Immunoglobulin-like domain
PF09085	Adhesion molecule, immunoglobulin-like
PF10648	Immunoglobulin-like domain of bacterial spore germination
PF08204	CD47 immunoglobulin-like domain
PF02832	Flavivirus glycoprotein, immunoglobulin-like domain
PF18667	Baseplate upper protein immunoglobulin like domain
PF08205	CD80-like C2-set immunoglobulin domain
PF17582	Cytomegalovirus UL20
PF08441	Integrin alpha
PF04729	ASF1 like histone chaperone
PF15005	Izumo sperm-egg fusion, Ig domain-associated
PF16167	Domain of unknown function (DUF4871)
PF00047	Immunoglobulin domain
PF13927	Immunoglobulin domain
PF13895	Immunoglobulin domain
PF16403	Domain of unknown function (DUF5011)
PF18705	Family of unknown function (DUF5643)
PF18452	Immunoglobulin domain
PF09099	Quinohemoprotein amine dehydrogenase, alpha subunit domain III
PF09100	Quinohemoprotein amine dehydrogenase, alpha subunit domain IV
PF11545	Cell surface heme-binding protein Shp
PF17622	Viral unique long protein 16
PF11589	Domain of unknown function (DUF3244)
PF07987	Domain of unkown function (DUF1775)
PF08527	Protein-arginine deiminase (PAD) middle domain
PF06328	Ig-like C2-type domain
PF17440	Thiol-activated cytolysin beta sandwich domain
PF18002	T6 antigen Ig like domain
PF13860	FlgD Ig-like domain
PF09261	Alpha mannosidase middle domain
PF08779	Betacoronavirus NS7A protein
PF16680	T-cell surface glycoprotein CD3 delta chain
PF09294	Interferon-alpha/beta receptor, fibronectin type III
PF14564	Membrane binding
PF17129	C-terminal domain of metallo-carboxypeptidase
PF16967	E-set like domain
PF09240	Interleukin-6 receptor alpha chain, binding
PF18368	Exo-beta-D-glucosaminidase Ig-fold domain
PF14888	Penicillin-binding protein Tp47 domain C
PF09291	Domain of unknown function (DUF1968)
PF00932	Lamin Tail Domain
PF09213	M3
PF09191	CD4, extracellular
PF20578	Atrophied bacterial Ig domain
PF14524	Wzt C-terminal domain

PF09094	Alpha-amylase/4-alpha-glucanotransferase, middle domain
PF01833	IPT/TIG domain
PF16158	Ig-like domain from next to BRCA1 gene
PF02480	Alphaherpesvirus glycoprotein E
PF16129	Domain of unknown function (DUF4841)
PF00554	Rel homology DNA-binding domain
PF18649	EcpB C-terminal domain
PF09134	Invasin, domain 3
PF18435	Esterase Ig-like N-terminal domain
PF16132	Domain of unknown function (DUF4843)
PF14686	Polysaccharide lyase family 4, domain II
PF16130	Domain of unknown function (DUF4842)
PF18962	Secretion system C-terminal sorting domain
PF18518	TcA receptor binding domain
PF13585	CHU_C Type IX secretion signal domain
PF18703	MALT1 Ig-like domain
PF09470	Telethonin protein
PF09167	Domain of unknown function (DUF1942)
PF07452	CHRD domain
PF18674	TarS beta-glycosyltransferase C-terminal domain 1
PF09259	Fungal immunomodulatory protein Fve
PF10425	C-terminus of bacterial fibrinogen-binding adhesin
PF18000	Type 2 DNA topoisomerase 6 subunit B C-terminal domain
PF16974	High-affinity nitrate transporter accessory, Ig-like domain
PF05689	Adhesion domain
PF10435	Beta-galactosidase, domain 2
PF02368	Bacterial Ig-like domain (group 2)
PF02369	Bacterial Ig-like domain (group 1)
PF02903	Alpha amylase, N-terminal ig-like domain
PF02927	Cellulase N-terminal ig-like domain
PF03723	Hemocyanin, ig-like domain
PF03785	Peptidase family C25, C terminal ig-like domain
PF07523	Bacterial Ig-like domain (group 3)
PF07532	Bacterial Ig-like domain (group 4)
PF10342	Kre9/KNH-like N-terminal Ig-like domain
PF10430	Tie-2 Ig-like domain 1
PF11614	IG-like fold at C-terminal of FixG, putative oxidoreductase
PF11940	Domain of unknown function (DUF3458) Ig-like fold
PF12245	Bacterial Ig-like domain
PF13205	Bacterial Ig-like domain
PF13750	Bacterial Ig-like domain (group 3)
PF14734	Domain of unknown function (DUF4469) with IG-like fold
PF16392	Ig-like domain
PF16640	Bacterial Ig-like domain (group 3)
PF17425	Arylsulfotransferase Ig-like domain
PF17433	Glycosyl hydrolase family 49 N-terminal Ig-like domain
PF18001	Interleukin-13 receptor subunit alpha Ig-like domain
PF18200	Bacterial Ig-like domain
PF19077	Bacterial Ig-like domain
PF19078	Bacterial Ig-like domain
PF19081	Ig-like domain CHU_C associated
PF20251	Bacterial Ig-like domain

PF20264	DUF4784 N-terminal Ig-like domain
PF00080	Copper/zinc superoxide dismutase (SODC)
PF02019	WIF domain
PF02221	ML domain
PF02246	Protein L b1 domain
PF02440	Adenovirus E3 region protein CR1
PF02494	HYR domain
PF05790	Immunoglobulin C2-set domain
PF07654	Immunoglobulin C1-set domain
PF07679	Immunoglobulin I-set domain
PF07686	Immunoglobulin V-set domain
PF08977	Bypass of Forespore C, N terminal
PF09118	Galactose oxidase-like, Early set domain
PF09136	Glucodextranase, domain B
PF10651	BppU N-terminal domain
PF11049	Glycoprotein K1 of Kaposi's sarcoma-associated herpes virus
PF11465	Natural killer cell receptor 2B4
PF11606	Family 31 carbohydrate binding protein
PF12988	TraQ conjugal transfer protein
PF14466	PLAT/LH2 and C2-like Ca2+-binding lipoprotein
PF15028	Pre-T-cell antigen receptor
PF15097	Immunoglobulin J chain
PF16650	Unstructured region on SPEG complex protein
PF16681	Ig-like domain on T-cell surface glycoprotein CD3 epsilon chain
